# The Synergy of School Climate, Motivation, and Academic Emotions: A Predictive Model for Learning Strategies and Reading Comprehension

**DOI:** 10.3390/bs15040503

**Published:** 2025-04-09

**Authors:** Panagiota Dimitropoulou, Diamanto Filippatou, Stamatia Gkoutzourela, Anthi Griva, Iouliani Pachiti, Michalis Michaelides

**Affiliations:** 1 Department of Psychology, University of Crete, 74150 Rethymno, Greece; psyp221@psy.soc.uoc.gr; 2 Department of Psychology, National and Kapodistrian University of Athens, 15784 Athens, Greece; filipd@psych.uoa.gr; 3 Greek Ministry of Education, Religious Affairs and Sports, 15180 Athens, Greece; matgoutz@sch.gr (S.G.); anthigriva@sch.gr (A.G.); 4 Department of Psychology, University of Cyprus, Nicosia 1678, Cyprus; michaelides.michalis@ucy.ac.cy

**Keywords:** school climate, academic emotions, motivation for reading, learning strategies, reading comprehension, self-determination theory, control–value theory, intrinsic motivation, extrinsic motivation, school psychology

## Abstract

This study examines the relationship between school climate, motivation, and academic emotions in shaping learning outcomes, with a focus on reading comprehension. Based on the control–value theory of academic emotions and self-determination theory, it explores how students’ perceptions of a caring school community and a positive learning climate influence their emotions, motivation, learning strategies, and reading performance. A total of 296 fifth- and sixth-grade students completed self-report measures assessing these factors. For the collection of the data, the participants completed the following scales: Motivation for Reading Questionnaire—MRQ, Achievement Emotions Questionnaire Pre-Adolescents (AEQ-PA), Learning Climate Questionnaire (LCQ), School as a Caring Community Profile-II-(SCCP-II), learning strategies questionnaire, and reading comprehension. The correlation and path analysis results revealed that students who viewed their school as supportive experienced more positive academic emotions and fewer negative ones. Positive emotions were linked to higher intrinsic and extrinsic reading motivation, while negative emotions correlated with lower motivation and disengagement. Intrinsic motivation significantly predicted deep learning strategies and improved reading comprehension, whereas the absence of learning strategies negatively impacted performance. These findings underscore the role of a supportive school environment in fostering motivation and emotional engagement. The educational implications of this study highlight the need for teaching practices that cultivate positive emotions, intrinsic motivation, and effective learning strategies to enhance reading comprehension and overall academic success.

## 1. Introduction

In recent years, there has been an increasing focus on the social and emotional dimensions of learning, as research continues to highlight the critical role of psycho-emotional factors in students’ adjustment and academic success (Schutz & Pekrun, 2007; Thapa et al., 2013; M.-T. Wang & Degol, 2016). Considerable attention has been directed toward the academic emotions experienced by both students and teachers within the school setting, the influence of school and classroom environments on student motivation, and the pivotal role of teachers in shaping classroom climate (Hagenauer et al., 2015; Pekrun et al., 2002; Putwain et al., 2013; Urdan & Schoenfelder, 2006). Moreover, ongoing research seeks to clarify how academic emotions contribute to the learning process by examining their relationship with motivational and cognitive factors that underpin academic achievement (Di Leo et al., 2019; L. Y. Jang & Liu, 2012; Mega et al., 2014; Obergriesser & Stoeger, 2020; Ranellucci et al., 2015).

Drawing upon previous empirical findings, the present study is grounded in a theoretical framework that delineates the interrelated roles of environmental, emotional, and cognitive variables in academic learning. Informed by self-determination theory and the control–value theory of academic emotions, the proposed model posits a sequential pathway in which students’ perceptions of a supportive school climate and classroom environment influence their academic emotions and reading motivation. These psychological constructs are, in turn, hypothesized to affect the use of learning strategies, ultimately shaping reading comprehension outcomes. By articulating this integrated framework, this study aims to contribute to a more comprehensive understanding of the mechanisms underlying reading achievement in educational contexts.

**Figure 1:**
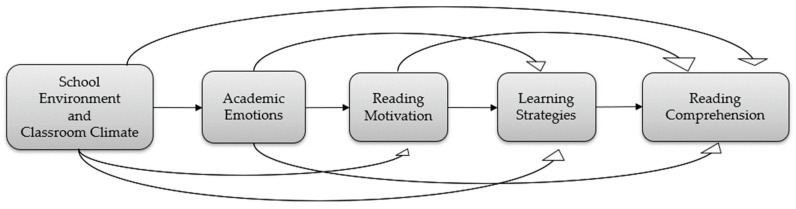
Theoretical pathway model of reading comprehension.

Accordingly, the following sections examine four core dimensions central to a proposed theoretical pathway model (see Figure 1): (1) academic emotions, (2) school and classroom climate, (3) reading motivation, and (4) learning strategies. Each is considered in relation to its role in influencing students’ cognitive engagement and academic outcomes, with particular emphasis on reading comprehension.

### 1.1. Academic Emotions and Learning Outcomes

The emotions experienced by students in the school or academic environment related to learning/academic activities and their outcomes are defined as academic emotions (Pekrun et al., 2002).

Students experience a wide range of emotions daily in academic settings, which can be triggered by activities such as reading a text (e.g., interest, boredom) (Ainley et al., 2005; Goetz et al., 2023) or taking an exam (e.g., satisfaction, shame) (Goetz et al., 2023; Turner & Schallert, 2001). Examples include the joy of learning, boredom during classroom instruction, and the frustration or anger that arises when facing difficult tests (Pekrun et al., 2006; Tze et al., 2014). Not all emotions that develop in educational contexts are academic; for instance, social emotions, such as a student caring for their classmates, also emerge. However, these two types of emotions often overlap, especially when reflecting on one’s feelings in relation to the performance of others (Pekrun et al., 2007).

Consistent with contemporary models of emotion dynamics, academic emotions are considered to be groups of interrelated psychological processes related to affective, cognitive, physiological, and motivational functions. For example, anxiety can include discomfort and intense feelings (affective function), worry (cognitive function), impulses to escape from a situation (motivational function), and physical symptoms (neurophysiological function) (Goetz et al., 2023; Pekrun et al., 2011; Scherer, 2009).

In this direction, the “control–value” theory of academic emotions (control value theory of achievement emotions) (Pekrun, 2000, 2019; Pekrun et al., 2002) provides a comprehensive framework for analyzing the effects of emotions experienced in educational settings. In particular, it assumes that emotions are related to achievement motivation and learning outcomes while also shaping key processes in the educational context. This theory is a multidimensional approach to analyzing a variety of emotions experienced in academic contexts as well as in achievement situations in other areas of life (sports, professional life) (Pekrun, 2024; Pekrun et al., 2011). The foundations of the theory, according to its authors, lie in hypotheses of theories about the expected value of emotions (Pekrun, 1992; Turner & Schallert, 2001), performance theories (Weiner, 1985), and models concerning emotions and their effects on achievement (Fredrickson, 2004; Pekrun, 1992; Zeidner, 2007). The theory extends these views by incorporating suggestions from different approaches and focuses on both emotions related to activities and emotions related to the final outcome of these activities (Pekrun et al., 2011).

In particular, Pekrun (2006) developed a three-dimensional classification of academic emotions. First, the division into emotions related to activities and emotions related to the outcome of these activities was performed based on the object of these emotions. A second classification was performed according to vigor, thus separating positive emotions from negative emotions. Finally, a third classification was performed according to the degree of activation since some emotions are considered to cause activation, such as hope, while others cause deactivation, such as despair. The above classification is consistent with complex models of emotion that place emotional states in a two-dimensional space (vigor x activation) (Linnenbrink, 2007).

According to Pekrun et al. (2007), “control” and “value” are the key concepts of the theory. Individuals experience specific emotions when they feel they have, or do not have, control over achievement activities and outcomes that are subjectively important to them. Ratings, i.e., “control” and “value”, are the proximate determinants of academic emotions. These evaluations, in turn, are influenced by factors as broad as the individual student’s goals and beliefs, as well as by temperament and genetic predispositions. They are also influenced by factors relating to classroom interaction, social environment, and the wider socio-historical context.

On the other hand, empirical evidence shows that academic emotions have a significant impact on students’ motivation, learning and achievement, personality development, and health (Schutz & Pekrun, 2007; Tan et al., 2021; Tyng et al., 2017). Positive emotions (joy, hope, pride) have been found to activate students’ motivation and academic achievement positively (Pekrun et al., 2002, 2004; Tan et al., 2021), while negative emotions (anger, anxiety, boredom) are associated with motivation avoidance and low levels of academic achievement (Daniels et al., 2009; Tan et al., 2021). According to recent research, students who experience positive academic emotions are more likely to achieve higher grades (Pekrun et al., 2011; Putwain et al., 2021; Villavicencio & Bernardo, 2013), pursue knowledge-oriented goals (Pekrun et al., 2006), and participate more actively in classroom activities (King & Gaerlan, 2014). Conversely, experiencing negative academic emotions leads students to decreased achievement (Pekrun et al., 2014) and reduced effort (Dettmers et al., 2011).

In conclusion, it appears that academic emotions affect academic engagement and performance, cognitive resources, motivation, strategies, self-determination or not to learn, and overall achievement. Finally, achievement, in turn, also affects students’ emotions and the environment inside and outside the classroom (Graziano et al., 2007).

### 1.2. The Role of the Classroom Environment in Motivation and Emotion

Classroom climate has been associated with a variety of outcomes for students and is therefore considered an important dimension. Although schools focus on measuring academic outcomes, psychosocial outcomes have been considered more important by some scholars (Babad, 2009; Rubie-Davies et al., 2016). Teacher behavior and practices have been found to be related to specific beliefs and, in particular, psychosocial beliefs have been found to influence classroom climate. Teachers’ beliefs about their self-efficacy, goals, and expectations are factors that influence both the teaching climate and the socio-emotional climate of the classroom and, therefore, the climate of the classroom in general (Rubie-Davies, 2014).

From the perspective of self-determination theory, learning climate is related to teachers being either autonomy-supportive or controlling. An autonomy-supportive teacher aims to thoroughly identify and support students’ interests, preferences, and emotions to encourage voluntary and enthusiastic participation in classroom learning activities. By creating a student-centered environment that aligns with individual interests, the teacher fosters intrinsic motivation, helping students become more actively and meaningfully engaged in the learning process. This approach empowers students to take ownership of their learning, making education both enjoyable and effective (Bureau et al., 2022; H. Jang et al., 2016; Reeve & Cheon, 2021; Ryan & Deci, 2020; Taylor et al., 2014). Many teachers display controlling, distant, or disorganized teaching styles (Aelterman et al., 2019; Van Den Berghe et al., 2013). In a controlling style, teachers push students to comply with their directives, disregarding student preferences, which undermines their sense of autonomy (Aelterman et al., 2019). Teachers with a cold style show little personal care or concern for students, hindering the students’ need for connection and relatedness (Van Den Berghe et al., 2013). A chaotic teaching style leaves students to manage on their own, often resulting in feelings of confusion or being overwhelmed, which weakens their sense of competence (Aelterman et al., 2019).

### 1.3. School as a Caring Community

There is still no consensus on the definition of a school community, nor is there extensive empirical research on the impact of school communities on teachers and students. Nevertheless, definitions of the community share some common elements (Battistich et al., 1997). According to Roberts et al. (1995), the term “community” focuses primarily on the quality of social relationships among school members. Conceptually, the term encompasses certain characteristics, such as members caring for, supporting, and assisting each other, actively participating and influencing decisions and activities, feeling a sense of belonging to a group with shared characteristics, and sharing common rules, goals, and values (Bryk & Driscoll, 1988; McMillan & Chavis, 1986).

Classrooms that function as caring communities are expected to have positive effects on students’ social, moral, and intellectual development. Students in such classrooms are anticipated to develop strong emotional bonds with one another and with their teachers, and this sense of identity and connection to the community is expected to motivate them to support community rules and internalize its values (Battistich et al., 1997).

The previous research progressed from the classroom level to the larger school community. The idea of the school as a “caring community” emerged in practice through intervention programs aimed at promoting prosocial behavior and social–emotional learning. This approach highlights the important role of the context (Battistich et al., 1997; Solomon et al., 1988). Overall, the concept of the school as a community provides a strong framework for examining educational practice and guiding educational reform efforts. Initially, Battistich et al. (1997) emphasized the sense of community experienced by students within their immediate classroom context, particularly because their study focused on elementary school students. However, they soon realized that to achieve sustainable positive outcomes, the sense of relatedness and belonging had to extend to the entire school community. The broader school context had to operate as a caring and supportive community.

Through the implementation of social–emotional learning programs, it became clear that the primary goal should be to create a community that addresses the fundamental psychological needs of students and teachers for autonomy, competence, and a sense of belonging. This also involves fostering values of justice, care, and responsibility for life in a democratic society (Battistich et al., 1997). Like other scholars, Battistich et al. (1997) hypothesized that students have basic psychological needs, including a sense of relatedness, autonomy, and competence, and that their engagement with the school is closely tied to whether these needs are met (Connell, 1990; Deci & Ryan, 1985). They further argued that these needs should be met at a group level, as the group provides individuals with recognition and enables them to commit, rather than offering mere individual support. The notion that students’ psychological needs are best met through active participation in a caring group with a shared purpose aligns with the concept of “community”, as defined by Battistich et al. (1997).

The coherence of a school has been found to play a role in the emotional and mental well-being of its members (Shochet et al., 2006). Recent research supports these earlier findings, providing new insights into the effects of caring school communities. For instance, the study by Urke et al. (2023) highlighted the longitudinal relationship between students’ perceptions of a caring school climate and their mental well-being, emphasizing the positive impact of supportive school environments.

There is ample evidence supporting the numerous benefits of schools that operate as communities for both students and teachers. Teachers who feel a sense of belonging to a community are more satisfied and effective in their work (Bryk & Driscoll, 1988). For students, the sense of community is associated with various positive outcomes, such as personal and social skills (e.g., conflict resolution, commitment to democratic values, empathy, and self-esteem), as well as academic-related variables like achievement motivation, intrinsic motivation for learning, school enjoyment, and reading comprehension (Arhar & Kromrey, 1993; Battistich et al., 1997; Bryk & Driscoll, 1988; Goodenow, 1993). The significance of the social context of schools for educational outcomes has been highlighted in contemporary educational and psychological research on school communities.

### 1.4. Reading Comprehension and Learning Strategies

Reading comprehension is a complex active process of constructing meaning from text through an interaction among automatic and strategic cognitive processes that enables the reader to create a mental representation of the text (Durkin, 1993; Kendeou et al., 2016; Van Den Broek & Espin, 2012). It depends not only on the reader’s characteristics, such as prior knowledge and working memory, but also on language processes, such as basic reading skills, decoding, vocabulary, and inferencing, as well as text characteristics (e.g., text structure, type of text, title, pictures, etc.). Comprehension also requires the effective use of strategic processes, such as metacognition and comprehension monitoring (Moore, 2015). Current theoretical models on reading comprehension point out the contribution of active learning strategies and skill use to reading comprehension (Cromley & Azevedo, 2007; Kintsch, 2009). More specifically, Cromley and Azevedo’s Direct and Inferential Mediation (DIME) Model (Cromley & Azevedo, 2007) describes reading comprehension as a combination of multiple skills, including background knowledge, vocabulary, reading strategies, and inference-making. The model emphasizes that reading comprehension directly depends on vocabulary and background knowledge, while inferences act as mediators. Additionally, reading fluency plays a crucial role—fluent readers decode words effortlessly, freeing cognitive resources for deeper text comprehension.

Learning strategies can be defined as any behavioral, cognitive, metacognitive, motivational, or affective process or action that facilitates understanding, learning, and meaningful encoding into memory. Research has identified three main groups of learning strategies: cognitive strategies, metacognitive strategies, and motivation and emotion regulation strategies (Dermitzaki, 2025; Weinstein & Mayer, 1986). Cognitive strategies involve the use of processing and organizational strategies to increase the encoding, retention, and comprehension of learning material. They include both surface strategies (e.g., repetition, process, memorization) and in-depth strategies (e.g., summary writing, questioning, etc.) (Alexander, 2004). Metacognitive strategies refer to the individual’s awareness of the use of cognitive strategies, such as planning, self-monitoring, and self-evaluation, to regulate the learning process (Flavell, 1979, 1987; Mokhtari & Reichard, 2002). Motivation and emotion regulation strategies play a role in managing self-motivation and anxiety (Dermitzaki & Papakosma, 2020; Efklides, 2011; Efklides & Schwartz, 2024; Lohbeck & Moschner, 2022; Nejadihassan & Arabmofrad, 2016). A wealth of research data demonstrates the positive contribution of cognitive and metacognitive strategies in organizing and assessing learning (Dinsmore, 2017; Pintrich, 2002; Swan, 2008) and in improving students’ performance (Droop et al., 2016; Elbro & Buch-Iversen, 2013; Schünemann et al., 2013). Considerable evidence indicates that reading proficiency and strategy use are related. In reading, strategies influence the way in which students select, acquire, organize, or integrate new knowledge (Dewulf et al., 2022; Pressley & Afflerbach, 1995; Weinstein et al., 2011). They play a significant role in reading comprehension since they turn readers into active self-regulated learners. Self-regulated readers are characterized by their intentional goal-setting for reading, active monitoring and regulation of their thoughts, strategic use of techniques to optimize meaning construction, and reflection on their reading behaviors in relation to their goals and results (Connor et al., 2016; Dinsmore, 2017; Efklides & Schwartz, 2024; Magi et al., 2016; Muijselaar et al., 2017; Sun et al., 2021).

In a meta-analysis examining the correlation between reading strategies and comprehension, it was found that the absence of specific strategies negatively impacts reading comprehension. The study synthesized data from 57 effect sizes, representing 21,548 readers, and concluded that reading strategies are crucial for effective comprehension (Sun et al., 2021). These results emphasize the importance of explicit reading strategy instruction in improving comprehension skills. Skilled readers tend to use more reading strategies than less proficient readers. Effective readers not only recognize which strategies to use but also understand when and where to apply them (Pressley & Gaskins, 2006). According to Zimmerman’s three-phase model of SRL (Zimmerman, 2000), good readers engage in strategic actions before, during, and after reading (Dermitzaki, 2025; Mason, 2013). Additionally, successful readers persist through reading difficulties and effectively motivate themselves throughout the process (Graham & Harris, 2018).

In addition, the use of reading strategies was found to be strongly tied to students’ learning motivation and self-efficacy (Dermitzaki & Papakosma, 2020; Moore, 2015; Spörer & Schünemann, 2014).

### 1.5. Integrating Environment, Emotion, and Cognition: Toward a Conceptual Model

An additional factor related to learning is motivation. Several studies have examined the reciprocal relationship between motivation, learning, and achievement (Kim & Pekrun, 2014; Schunk et al., 2010). According to Guthrie and Wigfield (2018), intrinsic motivation, self-efficacy, social motivation, value, and engagement include important motivational processes for reading, although engagement in reading appears to be the result of motivational processes as well (Wigfield & Guthrie, 2010). When learning is linked to intrinsic motivation, it happens for the sake of personal fulfillment and because learning is inherently interesting and enjoyable (Mouratidis & Michou, 2011). In their earlier studies, Wigfield and Guthrie referred to eleven dimensions of reading motivation (Guthrie et al., 1999; Wigfield & Guthrie, 1997). Intrinsic reading motivation included dimensions, such as curiosity, engagement, and challenge, while extrinsic motivation included dimensions, such as recognition and grades. The social dimension of reading was a separate dimension that was linked to achieving performance goals (competition). The concept of reading self-efficacy was considered a distinct theoretical construct.

According to self-determination theory, intrinsic motivation is related to the fulfillment of three basic psychological needs: competence, relatedness (feeling connected to others), and autonomy (feeling that one’s actions and pursuits are self-determined as opposed to controlled by others) both in academic and recreational settings (Ryan & Deci, 2000). This differs from extrinsic motivation, which involves performing tasks for the sake of earning rewards (e.g., good grades) or avoiding punishment (Hayenga & Corpus, 2010). Intrinsic motivation to learn has been associated with many positive academic outcomes (increased persistence in learning activities, fewer school dropouts, better retention and understanding of learning material) and manifold social–emotional benefits for students (more positive feelings toward homework, improved psychological well-being) (Froiland & Oros, 2014).

In particular, reading motivation affects reading behavior, i.e., the amount and frequency of reading, strategies, and preferences of the reader, as well as reading ability, i.e., skills, and comprehension (Guthrie et al., 2012). Internally motivated students read more for pleasure (J. H. Wang & Guthrie, 2004), use more and more complex learning strategies (Guthrie et al., 1996; Mokhtari & Reichard, 2002), and perform higher in reading comprehension (Retelsdorf et al., 2011; Stutz et al., 2016). In fact, the relationship between reading motivation and reading comprehension is thought to be bidirectional (Schiefele et al., 2012). On the one hand, intrinsically motivated readers are more likely to read voluntarily, practicing comprehension processes more extensively (Guthrie et al., 1999). At the same time, children who excel in reading tend to feel more confident and capable, which increases their intrinsic motivation, whereas children who struggle with reading are less intrinsically motivated (Chapman et al., 2000).

On the other hand, students’ extrinsic motivation is associated with the use of surface reading strategies (Schiefele et al., 2012), does not affect the amount of reading (Becker et al., 2010; Lau, 2009), or has negative effects on it (J. H. Wang & Guthrie, 2004). Extrinsically motivated children are likely to read only, when necessary, to perform well in school or to gain approval from their parents. Additionally, children who begin as poor readers often avoid reading due to the negative emotions they associate with it (Stanovich, 1986). Thus, these children tend to develop extrinsic motivation, reading primarily to obtain external rewards such as praise from others or good grades (Schiefele et al., 2016).

In addition, within the self-determination theory perspective, research revealed that recreational autonomous reading motivation is linked to more positive reading behaviors and improved reading comprehension (De Naeghel et al., 2012). However, in an academic context, only the corresponding association between autonomous reading motivation and the frequency of leisure-time reading could be confirmed. In other words, students dedicate more of their leisure time to reading, engage more deeply, pay greater attention to the text, and achieve higher scores in reading comprehension when they read for enjoyment or perceive it as personally meaningful, rather than when they feel internally or externally pressured to read during their free time. Interestingly, recreational controlled reading motivation showed a significant negative correlation with reading comprehension, suggesting that feeling pressured—either internally or externally—to read during leisure time is associated with lower reading comprehension performance.

In addition to the study of motivation and cognitive factors in relation to learning and achievement, particular emphasis has been placed on clarifying the role of academic emotions in the learning process. According to Pekrun’s cognitive and motivational model (Pekrun, 1992) and Pekrun’s control–value theory (Pekrun, 2006), the influence of academic emotions on the process and outcomes of learning depends on the mediating role of various cognitive and motivational mechanisms, with the role of motivation and learning strategies, cognitive resources, and the self-regulation of learning being of primary importance. Positive academic activating emotions, such as pleasure, facilitate self-regulation and the use of flexible learning strategies by increasing intrinsic motivation (Pekrun et al., 2002; Pekrun & Perry, 2014). On the other hand, positive academic deactivating emotions, such as relief and relaxation, can lead to superficial information processing (Pekrun et al., 2002), undermining current motivation while enhancing motivation to reconnect with the task in the future (Sweeny & Vohs, 2012).

Negative academic triggering emotions, such as anxiety, anger, and shame, are associated with the use of rigid learning strategies, such as repetition and memorization, and undermine the self-regulation of learning, prompting students to rely on external guidance from teachers and parents (Pekrun & Perry, 2014). The effects of enabling negative academic emotions on motivation vary. Specifically, anxiety and shame reduce intrinsic motivation but can trigger motivation for individuals to invest in future efforts to avoid failure (Pekrun et al., 2002; Pekrun & Perry, 2014). Negative academic deactivating emotions, such as hopelessness and boredom, involve cognitive deactivation, leading to reduced attention and more shallow information processing while reducing motivation to learn (Pekrun et al., 2002; Pekrun & Perry, 2014).

In terms of reading comprehension, drawing on the cognitive–motivational model of emotion effects within the control–value theory (Pekrun, 2006, 2019), it is anticipated that positive activating emotions, such as reading enjoyment, would help readers conserve cognitive resources, support the use of flexible reading strategies—such as elaborating on reading text and engaging in critical thinking—and encourage effort investment. Furthermore, it is expected that positive activating emotions enhance self-regulation, that is, setting flexible goals, planning, implementing reading strategies, and monitoring progress. Positive emotions tend to facilitate the incorporation of relevant items into long-term semantic memory during sentence comprehension (Pinheiro et al., 2013) and direct the reader’s attention to important features and information relevant to text comprehension, as well as to the use of deep processing and self-regulated strategies (Dermitzaki & Papakosma, 2020; Sedek & Von Hecker, 2004; Von Hecker & Meiser, 2005). On the contrary, positive deactivating emotions, such as relaxation, assurance, and relief, may temporarily reduce the motivation to exert effort in comprehending a text but can also enhance motivation to reengage in the task later (Pekrun et al., 2023).

Negative deactivating emotions, such as boredom and hopelessness could drain readers’ cognitive resources by triggering text-irrelevant thoughts and mind drifting, diminish motivation to read, and disrupt the structured use of comprehension strategies, ultimately resulting in superficial text information processing. Negative activating emotions, such as anxiety and shame, can lead to readers’ thoughts, such as worrying about not understanding the text. Additionally, they may diminish interest, intrinsic motivation to read a text, the use of flexible strategies, and self-regulation (Ellis et al., 1995, 1997; Zaccoletti et al., 2020). However, these emotions can also enhance extrinsic motivation to avoid failure, encourage the use of more rigid comprehension strategies, such as rehearsing learning material, and foster external regulation by increasing reliance on task assignments (i.e., on specific comprehension questions) and guidance from others (Pekrun et al., 2023).

Overall, research, although limited in terms of reading comprehension, reveals that emotions can facilitate or hinder the construction of mental representations of texts (Pekrun, 2022; Scrimin & Mason, 2015; Trevors et al., 2016, 2017). Apart from students’ cognitive and psycho-emotional variables, educational research increasingly acknowledges the significance of class-related factors on academic achievement (Dewulf et al., 2022). However, the interplay between class context, reading motivation, academic emotions, learning strategies, and reading comprehension remains poorly understood.

### 1.6. The Present Study: Testing a Theoretical Pathway Model

The present study aims to explore the interconnections between school as a caring community, learning climate, academic emotions, motivation towards reading, learning strategies, and reading comprehension. While previous studies have shown that academic emotions substantially affect student motivation and learning strategies (Pekrun et al., 2002, 2006), fewer studies have examined their direct and indirect effects on reading comprehension outcomes. Moreover, despite the extensively documented relationship between learning environments and students’ cognitive and emotional development (Battistich et al., 2000; Deci & Ryan, 2000), the underlying mechanisms remain underexplored.

By employing a path analysis model, we investigated the following hypotheses:

- **School Environment and Academic Emotions:** Students who perceive their school as a caring and supportive community and experience a positive learning climate will report higher positive academic emotions and lower negative academic emotions, aligning with theories emphasizing the role of supportive educational environments in emotional well-being and academic engagement (Battistich et al., 2000; Pekrun, 2006).
- **Academic Emotions and Motivation**: Positive academic emotions will be positively associated with both intrinsic and extrinsic reading motivation, while negative emotions will be linked to lower motivation levels. This is consistent with research suggesting that positive emotions enhance motivation and engagement, while negative emotions contribute to disengagement (Schunk & Zimmerman, 2012; Wigfield & Guthrie, 1997).
- **Motivation and Learning Strategies:** Intrinsic motivation will be positively related to the application of in-depth learning strategies, whereas extrinsic motivation will be more associated with surface learning strategies. Students with intrinsic motivation are more likely to engage in deep-level text processing, while extrinsically motivated students tend to adopt memorization-based approaches (Nolen & Haladyna, 1990; Ryan & Deci, 2000).
- **Learning Strategies and Reading Comprehension:** The use of in-depth learning strategies will be positively associated with higher reading comprehension scores, while the absence of learning strategies will negatively impact reading comprehension. This supports research indicating that effective strategy use enhances comprehension, whereas a lack of strategies impairs performance (Greensfeld & Nevo, 2017).

We also set the following exploratory research question:

- What are the direct and indirect pathways through which environmental, emotional, and motivational factors shape students’ reading comprehension?

Overall, the current research expands on prior findings by exploring the combined influence of environmental and psychological factors on reading comprehension, offering a more in-depth analysis of the key determinants of academic success among elementary school students.

## 2. Materials and Methods

### 2.1. Participants

The sample was composed of 296 elementary (grades 5 and 6) students (49.3% male) from 11 public schools in the prefecture of Attica. Overall, 49% were from the 5th grade and 51% were from the 6th grade. The students came from intact (85.8%), divorced (12.8%), or single-parent (1.4%) families. In terms of ethnicity, 30 (10.2%) students were of non-Greek ethnicity, but they had been attending the Greek school since the start of their formal education, and 16 respondents were diagnosed with learning difficulties (5.4%). More than half of the parents had graduated from university (52.9% of the fathers, 61% of the mothers), while regarding socio-economic status, most of the mothers worked (83.1%), and only 6 of the fathers (2.1%) were unemployed.

### 2.2. Procedure

Prior to data collection, consent was obtained from school the principals and teachers for the participation of their schools in this study. Subsequently, written parental consent was requested. The parents were informed about the purpose of this study through a written notice before providing their signed consent. Only the students with parental consent were eligible to participate. The administration of research instruments was conducted in a structured manner. All questionnaires were distributed collectively to the students in a single package, following a predetermined order. Instructions were provided for each questionnaire, and additional clarifications were given when requested. To ensure an unbiased response environment, the students were explicitly informed that there were no right or wrong answers, their responses would not be graded, and anonymity would be maintained.

To further minimize potential external influence, the classroom teacher was not present during the administration process. The researcher read each item aloud to ensure uniform comprehension. The total completion time was approximately two teaching hours. Additionally, the students were informed that they could withdraw from the questionnaire completion process at any time if they wished.

Upon completion, all questionnaires were collected by the researcher for further analysis. The study procedure was in line with the “Code on Ethics and Good Practice” of the Research Ethics Committee of the National and Kapodistrian University of Athens (NKUA).

### 2.3. Measurements

For the purpose of this study, the following questionnaires were distributed, covering psycho-emotional parameters, motivation, and learning:

**Motivation for Reading Questionnaire—MRQ** (Wigfield & Guthrie, 1997). The MRQ is the most commonly used measure of reading motivation (Mucherah & Yoder, 2008). The adaptation of the tool into the Greek language was developed by Dimitropoulou et al. (2016). Its 53 items are rated on a 4-point scale from “very different from me” to “a lot like me”. In total, the MRQ is composed of 11 constructs: (1) reading efficacy; (2) reading challenge; (3) work avoidance; (4) reading curiosity; (5) reading involvement; (6) importance of reading; (7) competition in reading; (8) reading recognition; (9) reading for grades; (10) social reasons for reading; and (11) reading compliances. However, since few of the above subscales had a satisfactory Cronbach’s alpha, only the composite subscales related to *intrinsic* and *extrinsic* motivation, which demonstrated a satisfactory or very good level of internal consistency, were used in this study. Three dimensions of the MRQ assess *intrinsic motivation*: reading curiosity (“I read to learn new information about what interests me”), reading involvement (“I make pictures in my mind when I read a text”), and importance of reading (“it is important for me to be good at reading”). The different types of *external motivation* assessed by the MRQ relate to the following dimensions: reading recognition (“my parents often tell me that I am good at reading”), reading for grades (“I read to improve my grades”), and competition in reading (“I try to give more correct answers than my friends to text questions”). Cronbach’s alpha for the internal motivation composite subscale (14 items) was 0.77, and for the external motivation composite subscale (15 items), it was 0.86 after removing items 23 and 34.

**Achievement Emotions Questionnaire Pre-Adolescents (AEQ-PA)** (Peixoto et al., 2015). The AEQ-PA scales were adapted from the AEQ (Pekrun et al., 2011). The adaptation of the tool into the Greek language was developed by Dimitropoulou et al. (2016) using the same method as described previously. It is a self-report instrument developed to measure the emotions of students in academic situations and comprises 48 items (6 items for each emotion) that assess class- and test-related emotions. The class version assesses boredom, hopelessness, anger, anxiety, enjoyment, and pride. The test version assesses the same emotions, except boredom, which is replaced by relief, i.e., a deactivating emotion. Items are answered on a 5-point Likert scale (completely disagree to completely agree). In the present study, we grouped the items for a general assessment of student emotions in the school environment according to their emotional state, namely, boredom, hopelessness, anger, anxiety, enjoyment, pride, and relief. Exploratory factor analysis was conducted with the maximum likelihood for dimension reduction. The Kaiser–Meyer–Olkin criterion was 0.697, and Bartlett’s test of sphericity was statistically significant. Two factors were extracted that explained a large proportion of the variance, i.e., 67.690%. The first factor comprised enjoyment, pride, and (negatively loaded) boredom emotions, and the second factor consisted of anxiety, hopelessness, anger, and relief items (see Table 1). The positive and negative emotion factors were negatively correlated at −0.346. Thus, we formed two variables for the path model: one for positive and one for negative academic emotions.

**The Learning Climate Questionnaire (LCQ)** (Williams & Deci, 1996). The questionnaire consists of 15 items answered on a Likert scale of 7 points from 1 (strongly disagree) to 7 (strongly agree), with an intermediate score of 4 (moderately agree). The adaptation of the tool into the Greek language was developed by Dimitropoulou and colleagues (Williams & Deci, 2012). It was designed for students to report how they perceive support from their instructors with regard to autonomy. The LCQ has a single underlying factor with high internal consistency (Williams & Deci, 1996). In the present study, the LCQ (sum of the 15 items) had Cronbach’s α 0.90.

**School as a Caring Community Profile-II-(SCCP-II) Questionnaire** (Dimitropoulou et al., 2012; Lickona & Davidson, 2012). The SCCP-II was designed to assess the degree to which students perceive school as a caring community. In the present study, the student’s version was used. SCCP-II was used for the Greek population in a study where exploratory factor analysis suggested five subscales (Lickona & Davidson, 2012). The questionnaire includes 42 items that provide the following dimensions: (a) Perceptions of Student Respect (e.g., students treat classmates with respect); (b) Perceptions of Student Friendship and Belonging (e.g., students help each other, even if they are not friends); (c) Perceptions of Students’ Shaping of their Environment (e.g., students try to have a positive influence on the behavior of other students); (d) Perceptions of Support and Care by and for the Staff (e.g., students can talk to their teachers about problems that are bothering them); and (e) Perceptions of Support and Care by and For Parents (e.g., teachers treat parents with respect). Students rate their agreement with statements about their school’s climate on a 5-point Likert scale (1 = rarely, 5 = almost always). For the present study, Cronbach’s alphas for the individual factors ranged from 0.75 to 0.86, while for the whole questionnaire was 0.92.

**Learning Strategies Questionnaire** (Gonida & Leontari, 2012). The learning strategies questionnaire assesses the strategies that students use during their studies. It includes 27 sentences organized into three subscales: (a) the scale that measures the absence of strategies (8 items) (e.g., While I am studying for a subject, I often feel like I do not know what it is referring to), (b) the use of high-level strategies, such as deep processing strategies, metacognitive strategies, and self-regulation strategies (14 items) (e.g., When I read a text, I make diagrams in which I connect the main points), and (c) the use of low-level strategies, such as surface strategies (5 items) (e.g., When I study, I try to memorize as much information as I can). Students were asked to rate the strategies they used on a 5-point Likert scale from 1 (strongly disagree) to 5 (strongly agree). In this study, sentence 11 was removed from the surface strategies. Cronbach’s α ranged from 0.61 to 0.83.

**Reading Comprehension** (Chrysochoou, 2006). Reading comprehension was assessed with a battery developed by Chrysochoou (Chrysochoou, 2006; Chrysochoou et al., 2011; see also Chrysochoou & Bablekou, 2011) in the absence of standardized tests in the Greek language, providing measures of higher-order comprehension skills. The participants were presented with 5 written stories—one of which was used for practice—accompanied by 10 open-ended questions tapping 4 higher-order comprehension skills: generation of necessary and elaborative inferences, simile comprehension, and comprehension control. Stories and part of the questions were drawn from Oakhill (Oakhill, 1984) and Cain and Oakhill (Cain & Oakhill, 1999). Extra questions were added, and the stories were adapted for use with Greek children. The students were asked to read each text in a given time frame and then answer the questions on the next page in a short way, without turning the page and looking at the text. They were given eight minutes for each text, at the end of which the researcher asked the students to move on to the next text. The order of the stories was different for each student.

The above questionnaires were used in studies that have been conducted in Greece before, and their factorial structure was confirmed for the Greek population (Hatzichristou et al., 2020). Finally, demographic data were also collected related to the gender of the students, their grade of attendance, and their origin, place of birth, and number of siblings. Information was also recorded on the marital and occupational status of the parents, as well as their educational level.

### 2.4. Statistical Analysis

The statistical analysis of the dataset involved the computation of descriptive statistics, correlations of the variables, and factorial analyses. To study the direct and indirect effects between the variables, a path analysis was used. The data analysis was conducted using SPSS 23.0 (IBM Corp., 2015) and SPSS AMOS 22.0 (Arbuckle, 2013). The model examined empirically was a saturated model, as can be seen in Figure 1. The examination of assumptions for the path analysis included tests for non-normality and multicollinearity. Due to the large sample size, the Kolmogorov–Smirnov tests for the endogenous variables were significant. However, the extent of non-normality was not large: the skewness and kurtosis values never exceeded one in absolute value, so no transformations were applied to the variables. Bivariate correlations between the variables were, in most cases, low to moderate. The largest correlation was 0.679 for SURFACE-LS and DEPTH-LS, so there was no concern for multicollinearity. There were no missing data in the observed variables of the path analysis model.

## 3. Results

### 3.1. Descriptive Statistics and Correlations

Table 2 presents the descriptive statistics and correlations between the study variables. The two environmental variables (SCC and LC) showed a moderate positive correlation (r = 0.607, *p* = 0.000), indicating that students who perceive their school as a caring community are also likely to experience a more positive learning environment in the classroom. Both SCC and LC had significant moderate positive correlations with PAEm and weak positive correlations with intrinsic and extrinsic motives for reading. Conversely, they showed weak negative correlations with NAEm. These findings suggest that students who perceive their school as a caring community and feel supported by an enriching learning climate experience more positive emotions, exhibit higher motivation for reading, both for personal satisfaction and external rewards, and tend to experience fewer negative emotions about school. Additionally, these students are more likely to adopt constructive learning strategies, as evidenced by the positive correlations with SURFACE-LSs and DEPTH-LSs. At the same time, both SCC and LC demonstrated weak but significant negative correlations with ABSENCE-LSs, indicating that students in more supportive academic environments are less likely to lack structured learning strategies. Furthermore, only LC exhibited a weak but significant positive correlation with RC, suggesting that a supportive learning climate may slightly enhance students’ reading comprehension abilities. These findings imply that while both SCC and LC positively influence students’ emotions, motivations, and learning strategies, the specific perception of a positive classroom climate uniquely contributes to reading comprehension outcomes.

Regarding academic emotions, PAEms and NAEms showed a significant negative correlation with each other, as expected. PAEms had moderate positive correlations with both IMR and EMR (r = 0.490, *p* = 0.000, and r = 0.666, *p* = 0.000, respectively), suggesting that students who experience positive emotions in school are more motivated to read, both for personal interest and external rewards. Additionally, PAEms were positively associated with SURFACE-LSs and DEPTH-LSs, implying that students with positive emotions tend to adopt both low-level and high-level learning strategies. However, PAEms did not show a significant correlation with ABSENCE-LSs or RC.

Conversely, NAEms were negatively correlated with IMR, EMR, SURFACE-LSs, and DEPTH-LSs showing that negative academic emotions are associated with decreased motivation to read and reduced use of both low-level and high-level learning strategies. Moreover, NAEms had a positive correlation with ABSENCE-LSs, indicating that students who experience more negative emotions are more likely to lack effective learning strategies. NAEms also showed a negative correlation with RC, suggesting that students who experience more negative academic emotions are more likely to struggle with reading comprehension.

In terms of motivation for reading, IMR and EMR displayed a moderate positive correlation (r = 0.481, *p* = 0.000), implying that students who are motivated to read for personal enjoyment could also be driven by external rewards. Both IMR and EMR were positively associated with SURFACE-LSs and DEPTH-LSs. However, neither IMR nor EMR was significantly correlated with ABSENCE-LSs. These results suggest that students who are motivated to read, whether intrinsically or extrinsically, tend to employ both low-level and high-level learning strategies. With regard to academic performance, IMR showed a weak positive correlation with RC, suggesting that intrinsically motivated students tend to perform slightly better in reading comprehension.

Finally, regarding learning strategies, SURFACE-LSs were strongly and positively correlated with DEPTH-LSs (r = 0.679, *p* = 0.000), indicating that students may employ both low-level and high-level learning strategies simultaneously. Both SURFACE-LSs and DEPTH-LSs were negatively correlated with the absence of learning strategies, as expected. In terms of academic performance, only the use of high-level learning strategies (DEPTH-LSs) showed a weak positive correlation with RC, suggesting that students who engage in deep learning strategies tend to perform slightly better in reading comprehension. In contrast, the absence of using learning strategies was negatively correlated with RC, indicating that students who lack structured learning strategies tend to perform worse in reading comprehension.

### 3.2. Path Analysis Model

A path analysis was conducted to examine the direct and indirect effects between the variables in the model. For this purpose, a saturated model was employed, where all possible paths among variables were specified, resulting in zero degrees of freedom. This means that the model perfectly fits the data, as it reproduces the observed covariance matrix exactly. Furthermore, for the purposes of this study, (a) the school environment consisted of two major composite variables: school as a caring community (SCC) and learning climate (LC), (b) academic emotions were analyzed as positive (PAEms) and negative emotions (NAEms), (c) reading motivation was composed of intrinsic motives (IMR) and extrinsic motives (EMR), and (d) learning strategies included high-level strategies, such as in-depth strategies (DEPTH-LSs), low-level strategies, such as surface strategies (SURFACE-LSs), and the absence of strategies (ABSENCE-LSs). In particular, this study examined whether there is a direct or indirect effect between the environment (SCC and/or LC) and reading comprehension (RC) or whether their relationship is mediated by emotions, reading motives, and learning strategies.

#### 3.2.1. Direct Effects of the Variables

**Figure 2:**
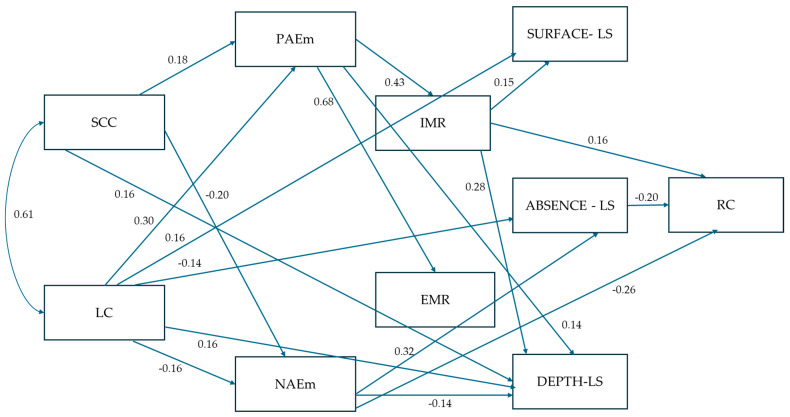
Path diagram of the saturated model tested with standardized regression coefficients. Note 1. Solid arrows indicate significant effects. Non-significant effects are omitted. Error variances and error covariances between subscales of an instrument are not shown on the diagram for clarity. Note 2. SCC = school as a caring community; LC = learning climate; PAEms = positive academic emotions; NAEms = negative academic emotions; IMR = intrinsic motives of reading; EMR = extrinsic motives of reading; DEPTH-LSs = in-depth learning strategies; SURFACE-LSs = surface learning strategies; ABSENCE-LSs = absence of learning strategies; RC = reading comprehension.

The path analysis revealed several significant direct effects among the variables, as shown in Figure 2. All effects and associated statistics appear in Appendix A, Table A1. The SCC environment demonstrated a significant positive direct effect on PAEms and a negative direct effect on NAEms. Similarly, the LC environment significantly influenced both PAEms and NAEms. Both SCC and LC environments had significant direct effects on the use of high-level learning strategies (DEPTH-LSs). The LC environment positively affected the use of low-level learning strategies and negatively impacted the absence of using learning strategies. In contrast, the SCC environment did not have a significant effect on these variables. Neither the SCC nor the LC environments showed significant direct effects on the motivation variables or reading comprehension.

Regarding academic emotions, PAEms had significant positive direct effects on both IMR and EMR, while NAEms did not significantly impact either. PAEms significantly affected the use of high-level strategies, but not the absence of learning strategies or the use of low-level learning strategies. Conversely, NAEms had a significant negative direct effect on DEPTH-LSs and a positive effect on ABSENCE-LSs. NAEms negatively affected RC, but PAEms did not show a direct effect on RC.

Intrinsic and extrinsic reading motives significantly influenced learning strategies. More specifically, IMR positively impacted both SURFACE-LSs and DEPTH-LSs. Conversely, EMR did not significantly affect the use of any type of learning strategies. IMR had a positive direct effect on RC, while EMR did not. Neither the use of high-level (DEPTH-LSs) nor low-level (SURFACE-LSs) learning strategies directly impacted reading comprehension. However, the absence of using learning strategies negatively influenced RC. 

#### 3.2.2. Indirect Effects of the Variables

In examining the indirect effects within the model, several notable findings emerged (see Appendix A, Table A2, for the full results regarding indirect effects along with 90% bootstrap confidence intervals). IMR and EMR were influenced indirectly by SCC (0.065 and 0.109, respectively) and LC (0.057 and 0.104, respectively), indicating a mediated effect from the two environmental variables via academic emotions. Significant indirect effects were also found for high-level and low-level learning strategies (DEPTH-LSs and SURFACE-LSs) from SCC, LC, and PAEms. No indirect effects were found for the absence of learning strategies. Finally, reading comprehension (RC) was indirectly positively influenced by SCC and negatively by NAEms.

## 4. Discussion

This study aimed to examine how environmental, psycho-emotional, and cognitive variables interact to shape students’ reading comprehension outcomes. Grounded in self-determination theory and the control–value theory of academic emotions, we tested a theoretical model proposing that students’ perceptions of a caring and autonomy-supportive school environment influence their academic emotions. These emotions—both positive and negative—subsequently shape their intrinsic and extrinsic motivation to read, which affects the learning strategies they employ and, ultimately, their reading comprehension performance. The results of the correlations, as well as the direct and indirect effects derived from the path analysis, are discussed in detail below. The findings offer empirical support for this multi-step pathway and highlight both direct and mediated effects across components of the model.

### 4.1. School Environment and Academic Emotions

One of the first findings was the strong positive association between school as a caring community and learning climate. This aligns with M.-T. Wang and Degol’s (2016) review, which emphasized that a supportive and inclusive school environment fosters a more positive learning climate. Additionally, school as a caring community and learning climate were positively associated with positive academic emotions and negatively associated with negative academic emotions. This finding is supported by previous studies, including the broaden-and-build theory of positive emotions (Fredrickson, 2001), which suggests that supportive environments enhance students’ emotional well-being and foster adaptive academic behaviors. Additionally, recent research by Chen et al. (2025) demonstrated that a perceived positive school climate is associated with reduced negative emotions among students.

### 4.2. Academic Emotions and Motivation for Reading

With respect to academic emotions and motivation, in the present study, positive academic emotions had a strong positive correlation with both intrinsic and extrinsic motives of reading. This is aligned with the control–value theory of academic emotions (Pekrun, 2006), which suggests that positive emotions enhance intrinsic and extrinsic motivation (King & Gaerlan, 2014; Tan et al., 2021). In addition, negative academic emotions were negatively related to intrinsic and extrinsic motives of reading. The latter is corroborated by Mega et al. (2014), who found that negative emotions, such as anxiety and frustration, can undermine motivation and contribute to disengagement.

### 4.3. Reading Motivation, Learning Strategies, and Reading Comprehension

Regarding motivation, learning strategies, and reading comprehension, intrinsic reading motivation was positively correlated with in-depth learning strategies, a finding consistent with the existing literature. For example, Šabec (2022) identified several factors contributing to students’ success in reading comprehension, emphasizing the role of intrinsic motivation and the implementation of effective reading strategies in primary school classrooms. Conversely, extrinsic reading motivation was positively correlated with both in-depth learning strategies and surface learning strategies, aligning with Deci and Ryan’s (2000) self-determination theory, which suggests that extrinsic motivation can influence both deep and surface learning behaviors depending on the learner’s perceived autonomy.

The negative correlation between the absence of learning strategies and reading comprehension supports previous research indicating that students who do not utilize structured learning strategies often struggle with reading comprehension tasks (Pintrich, 2004). Additionally, the weak yet significant positive relationship between deep learning strategies and reading comprehension is consistent with studies suggesting that deep learning strategies can improve comprehension, though their effectiveness may be influenced by other cognitive and metacognitive factors (Magnusson et al., 2019).

Finally, a notable finding was the weak correlation between extrinsic motives of reading and reading comprehension, indicating that external rewards alone may not substantially improve reading comprehension. This finding is in accordance with previous research suggesting that while extrinsic motivation can promote engagement, it is generally less effective in facilitating deeper cognitive processing (Guthrie et al., 2012). On the other hand, the positive correlation between intrinsic motives of reading and reading comprehension supports the idea that intrinsic motivation fosters deeper interactions with reading materials, ultimately enhancing comprehension outcomes (Schiefele, 2009).

### 4.4. Direct and Indirect Effects of Environmental and Psychological Variables

To detect direct and indirect effects, a path analysis was conducted among the variables. The results revealed a direct positive effect between the learning environment and positive academic emotions, as well as a direct negative effect with negative academic emotions, aligning with the relevant literature. A classroom environment that fosters personal competence, autonomy, and positive relationships appears to be essential for effective learning (M.-T. Wang & Holcombe, 2010). Furthermore, Fong Lam et al. (2015) emphasized the importance of considering school-based environmental factors that influence learning, as positive emotions, such as enjoyment, hope, and pride, are more likely to arise when students perceive their learning environment positively. Greensfeld and Nevo (2017) further argued that positive and negative emotions play a crucial role in learning processes, as students’ emotional reactions to academic tasks can shape their cognitive engagement and persistence (González & Paoloni, 2014; Paoloni, 2014). Conversely, Anttila et al. (2018) noted that conflicts can arise when students lose interest in learning or feel unsupported by their teachers, often leading to feelings of incompetence, disappointment, frustration, boredom, or fatigue.

Additionally, research indicates that students’ perceptions of the learning environment are closely linked to affiliation and extrinsic motivation (Wei & Elias, 2011). These perceptions significantly influence cognitive processes and academic outcomes and play a key role in fostering student motivation and cognitive development (Abolmaali & Mahmudi, 2013).

Consistent with our hypothesis, the results of the present study indicated that intrinsic motivation directly influenced the use of surface and deep learning strategies as well as the reading comprehension outcomes. These results are in line with previous research that consistently confirms the positive association between intrinsic motivation and proficiency in reading comprehension, even when controlling for a variety of relevant cognitive factors (Schiefele et al., 2012). This finding exists in samples from preschool to high school grades (Guthrie & Wigfield, 2000; Logan et al., 2011; Morgan & Fuchs, 2007; Retelsdorf et al., 2011; Taboada et al., 2009; Toste et al., 2020). More specifically, highly motivated students seem to demonstrate increased goal-directed actions and engagement (Vansteenkiste et al., 2006), are determined and focused despite challenges (Multon et al., 1991; Schunk, 1991), and spend more time reading (Toste et al., 2020).

Regarding the influence of intrinsic and extrinsic reading motivation on learning strategies, it is evident in the relevant research (Liao et al., 2022) that more intrinsically motivated students not only tended to use more strategies (Pardo, 2004) but also tended to use higher-level reading strategies (e.g., integrating text contents with elaborations) (McDaniel & Einstein, 2000; Naceur & Schiefele, 2005). They had a stronger intention and desire to understand the meaning of texts via deep-level text processing than less-interested students who were more inclined to process and store verbatim text features (Meece et al., 1988; Nolen & Haladyna, 1990; Schiefele, 1990). Actually, in the present study, intrinsic motivation positively influenced both surface and deep learning strategies, reinforcing the idea that students employ a combination of memorization-, elaboration-, and comprehension-based strategies to understand texts. It seems that Greek students in grades 5 and 6 need to use a range of strategies that include both memorization and repetition of information, as well as questioning, summarizing, predicting, making diagrams, etc., in order to understand what they read. To provide further clarity on this result, we should note that in the Greek Educational System, even in grades 5 and 6, students’ comprehension assessment includes memorization questions as well, apart from inference and critical thinking. In fact, employing a variety of strategies can help students improve their comprehension (Muijselaar et al., 2017; Samuelstuen & Bråten, 2005).

Moreover, the findings reveal that the absence of learning strategies negatively impacts reading comprehension. Students who do not develop strategic reading approaches tend to struggle with decoding and understanding texts, leading to decreased comprehension. This finding implies that the absence of effective reading strategies may significantly impair reading comprehension. For instance, poor readers typically do not read strategically, lack sufficient metacognitive awareness, and may have difficulty decoding words, leading to reduced comprehension (Sun et al., 2021).

In addition to the direct effects, the model also revealed important indirect pathways that underscore the complex interactions between the school environment, academic emotions, learning strategies, and reading comprehension. The school as a caring community and the learning climate had an indirect impact on intrinsic and extrinsic motives of reading through positive and negative academic emotions, which underscores the mediating role of academic emotions in shaping students’ motivation. These findings align with previous research suggesting that students’ perceptions of a supportive school climate foster psychological engagement, which subsequently influences their academic motivation and behavior (Maxwell et al., 2017).

The role of positive academic emotions in influencing learning was further reinforced by their indirect effects on deep learning strategies, aligning with previous research demonstrating that positive emotions enhance deeper engagement with learning materials (Zahid, 2014). In contrast, negative academic emotions exhibited minimal indirect effects on deep learning strategies, suggesting that their impact is primarily exerted through direct pathways rather than through other variables. This implies that while negative emotions directly undermine the use of learning strategies and comprehension, they do not significantly affect other factors that contribute to reading achievement through strategy use.

### 4.5. Summary and Limitations

In summary, reading comprehension was indirectly influenced by both schools as a caring community and learning climate and by intrinsic reading motivation, emphasizing the varied pathways through which environmental and psychological factors contribute to reading development. Specifically, a positive school climate enhances comprehension by fostering positive academic emotions and intrinsic motivation. These findings are consistent with previous research suggesting that student engagement and emotional experiences play a crucial role in linking school climate to academic success (W. Wang et al., 2014).

Conversely, reading comprehension was negatively affected by negative academic emotions, indicating that negative emotions may weaken comprehension through complex interactions with other variables. This aligns with prior studies showing that extrinsically motivated students often prioritize performance-oriented goals over deep learning, which can result in poorer comprehension outcomes (Goddard et al., 2000).

While this study offers valuable insights, it is not without limitations. First, the reliance on self-report questionnaires may introduce response bias. To enhance the validity of the findings, future research should incorporate objective measures of reading comprehension, such as standardized assessments or other updated techniques. Second, this study was conducted in a limited number of schools within a specific educational context in Greece, which may restrict its applicability to other cultural settings. Conducting cross-cultural comparisons could provide a broader understanding of how school climate, emotions, and motivation interact across diverse educational systems.

Additionally, longitudinal studies are necessary to explore causal relationships among the identified variables. Future research should also investigate the impact of digital learning tools and artificial intelligence on reading motivation and strategy use, considering the growing role of technology in education.

## 5. Concluding Remarks and Implications for Practice

This study examined how environmental factors, psycho-emotional variables, and cognitive processes interact to influence students’ academic experiences. The findings provide empirical support for the control–value theory of academic emotions (Pekrun, 2006) and self-determination theory (Deci & Ryan, 2000). The results reaffirmed the importance of a positive school climate in establishing positive academic emotions, increased motivation, and improved reading comprehension. A noteworthy observation was that when students perceived their school as a caring and supportive community, positive academic emotions, such as enjoyment and confidence, increased along with a decrease in negative emotions, such as anxiety and frustration.

Additionally, intrinsic reading motivation was strongly linked to the use of deep learning strategies. These strategies, which involve critical thinking and meaningful engagement with texts, significantly contributed to better reading comprehension. In contrast, extrinsic motivation showed a weaker connection to comprehension. While external incentives may encourage students to read, they do not necessarily lead to deeper cognitive engagement with the material.

This study also highlighted the importance of structured learning strategies. Students who lacked effective reading strategies tended to struggle with comprehension, reinforcing the need for explicit instruction in both deep and surface learning approaches. Moreover, the findings suggest that both types of strategies contribute to comprehension.

Further analysis revealed that school climate indirectly influences reading comprehension by shaping students’ academic emotions and motivation. Positive emotions facilitated the use of deep learning strategies, which, in turn, enhanced comprehension, whereas negative emotions had a direct negative impact on both learning strategies and reading outcomes. Intrinsic motivation emerged as a key driver of reading success, directly influencing both learning approaches and comprehension performance.

These findings underscore the necessity for educational practices that not only cultivate positive academic emotions and intrinsic motivation but also strategically incorporate effective learning strategies to optimize reading comprehension.

In particular, for educators, fostering a supportive classroom environment enhances student motivation and engagement. Encouraging intrinsic motivation—by designing reading activities that spark curiosity—promotes deeper cognitive engagement, rather than relying on external incentives. Additionally, explicit reading strategy instruction should be embedded in teaching to equip students with both deep- and surface-level comprehension techniques, enhancing their learning outcomes.

Furthermore, for policymakers, the findings highlight the need for holistic educational policies that consider students’ emotional and motivational well-being alongside cognitive skills. Professional development programs could train educators in social–emotional learning (SEL) strategies, fostering resilience and motivation.

In terms of the school community, a caring and inclusive school climate is essential for both academic success and student well-being. Strengthening collaboration between teachers, parents, and school psychologists ensures emotional and academic support for students, particularly those experiencing reading-related anxiety. Structured interventions for struggling readers should address both cognitive and emotional barriers to learning, promoting a more positive relationship with reading.

## Figures and Tables

**Table 1:** Exploratory factor analysis loadings (pattern matrix) of the seven academic emotion items.

Items	Positive Emotions	Negative Emotions
Enjoyment	1.017	
Pride	0.771	
Boredom	−0.524	
Anxiety		0.863
Hopelessness		0.804
Anger		0.530
Relief		0.520

**Table 2:** Descriptive statistics and correlations for the variables in this study.

Variables	#Items	Mean	SD	2	3	4	5	6	7	8	9	10
SCC	42	3.46	0.60	0.607 **	0.365 **	−0.297 **	0.256 **	0.220 **	0.286 **	0.417 **	−0.178 **	0.055
2. LC	15	5.22	1.08		0.411 **	−0.283 **	0.281 **	0.285 **	0.339 **	0.429 **	−0.199 **	0.116 *
3. PAEms	6	6.77	1.05			−0.408 **	0.490 **	0.666 **	0.385 **	0.460 **	−0.054	−0.012
4. NAEms	16	3.91	1.02				−0.249 **	−0.225 **	−0.237 **	−0.357 **	0.318 **	−0.284 **
5. IMR	14	3.08	0.45					0.481 **	0.339 **	0.467 **	−0.029	0.119 *
6. EMR	15	3.05	0.57						0.326 **	0.342 **	0.020	−0.066
7. SURFACE-LSs	4	3.82	0.69							0.679 **	−0.219 **	0.039
8. DEPTH-LSs	14	3.87	0.56								−0.172 **	0.117 *
9. ABSENCE-LSs	8	2.39	0.74									−0.275 **
10. RC	10	25.27	6.39									

* *p* < 0.05 and ** *p* < 0.01. Note. SCC = school as a caring community; LC = learning climate; PAEms = positive academic emotions; NAEms = negative academic emotions; IMR = intrinsic motives of reading; EMR = extrinsic motives of reading; DEPTH-LSs = in-depth learning strategies; SURFACE-LSs = surface learning strategies; ABSENCE-LSs = absence of learning strategies; RC = reading comprehension.

## Data Availability

The data are available in a publicly accessible repository: OSF link https://osf.io/wbz79/?view_only=e9a931665d8044b3b654fa116e5af5e7 (accessed on 13 February 2025).

## Appendix A

**Table A1:** Direct effects.

Predictor	On Outcome Variable	b	S.E.	Beta	Critical Ratio	*p*
*Direct effects of learning environments*						
School as a caring community	Positive emotions	0.322	0.116	0.183	2.779	0.005
	Negative emotions	−0.337	0.118	−0.197	−2.848	0.004
	Intrinsic read. motivation	0.036	0.049	0.048	0.742	0.458
	Extrinsic read. motivation	−0.038	0.053	−0.04	−0.719	0.472
	Surface LS	0.073	0.076	0.064	0.961	0.336
	Absence LS	−0.067	0.086	−0.054	−0.777	0.437
	Depth LS	0.145	0.055	0.156	2.633	0.008
	Reading comprehension	−0.929	0.738	−0.087	−1.259	0.208
Learning climate	Positive emotions	0.293	0.065	0.299	4.539	<0.001
	Negative emotions	−0.155	0.066	−0.163	−2.358	0.018
	Intrinsic read. motivation	0.027	0.028	0.064	0.972	0.331
	Extrinsic read. motivation	0.023	0.03	0.043	0.764	0.445
	Surface LS	0.101	0.043	0.158	2.354	0.019
	Absence LS	−0.097	0.049	−0.141	−1.991	0.047
	Depth LS	0.082	0.031	0.158	2.638	0.008
	Reading comprehension	0.596	0.419	0.101	1.423	0.155
*Direct effects of academic emotions*						
Positive emotions	Intrinsic read. motivation	0.184	0.025	0.429	7.277	<0.001
	Extrinsic read. motivation	0.37	0.027	0.685	13.529	<0.001
	Surface LS	0.091	0.051	0.14	1.79	0.073
	Absence LS	0.071	0.058	0.1	1.224	0.221
	Depth LS	0.077	0.037	0.145	2.081	0.037
	Reading comprehension	−0.873	0.494	−0.144	−1.766	0.077
Negative emotions	Extrinsic read. motivation	0.031	0.027	0.055	1.141	0.254
	Intrinsic read. motivation	−0.018	0.025	−0.041	−0.739	0.46
	Surface LS	−0.039	0.039	−0.058	−1.009	0.313
	Absence LS	0.236	0.044	0.325	5.361	<0.001
	Depth LS	−0.075	0.028	−0.137	−2.676	0.007
	Reading comprehension	−1.602	0.393	−0.257	−4.072	<0.001
*Direct effects of reading motivation*						
Intrinsic reading motivation	Surface LS	0.233	0.094	0.153	2.493	0.013
	Depth LS	0.342	0.068	0.277	5.064	<0.001
	Absence LS	0.039	0.106	0.024	0.369	0.712
	Reading comprehension	2.278	0.934	0.161	2.438	0.015
Extrinsic reading motivation	Depth LS	0.002	0.062	0.002	0.03	0.976
	Surface LS	0.106	0.087	0.088	1.229	0.219
	Absence LS	0.087	0.098	0.067	0.886	0.376
	Reading comprehension	−1.199	0.832	−0.107	−1.44	0.15
*Direct effects of learning strategies*						
Absence of learn. strategies	Reading comprehension	−1.693	0.5	−0.197	−3.387	<0.001
Depth learning strategies	Reading comprehension	0.813	0.941	0.071	0.864	0.387
Surface learning strategies	Reading comprehension	−0.8	0.689	−0.086	−1.161	0.246

**Table A2:** Indirect effects.

Predictor	On Outcome Variable	b	S.E.	Beta	90% Lower CI	90% Upper CI
*Indirect effects of learning environments*						
School as a caring community	Extrinsic read. motivation	0.109	0.045	0.047	0.036	0.183
	Intrinsic read. motivation	0.065	0.026	0.034	0.027	0.114
	Depth LS	0.085	0.033	0.034	0.037	0.146
	Absence LS	−0.046	0.032	0.026	−0.109	−0.001
	Surface LS	0.074	0.035	0.03	0.022	0.139
	Reading comprehension	0.665	0.442	0.04	0.014	1.489
Learning climate	Extrinsic read. motivation	0.104	0.026	0.045	0.061	0.15
	Intrinsic read. motivation	0.057	0.015	0.033	0.035	0.084
	Depth LS	0.063	0.017	0.032	0.037	0.094
	Absence LS	−0.002	0.022	0.031	−0.037	0.036
	Surface LS	0.066	0.021	0.031	0.035	0.105
	Reading comprehension	0.183	0.243	0.041	−0.175	0.625
*Indirect effects of academic emotions*						
Positive emotions	Depth LS	0.064	0.023	0.044	0.026	0.103
	Absence LS	0.039	0.036	0.051	−0.019	0.099
	Surface LS	0.082	0.035	0.053	0.023	0.139
	Reading comprehension	−0.236	0.341	0.057	−0.778	0.348
Negative emotions	Depth LS	−0.006	0.01	0.019	−0.025	0.009
	Absence LS	0.002	0.007	0.009	−0.007	0.015
	Surface LS	−0.001	0.01	0.015	−0.018	0.015
	Reading comprehension	−0.515	0.166	0.026	−0.783	−0.258
*Indirect effects of reading motivation*						
Intrinsic reading motivation	Reading comprehension	0.025	0.355	0.025	−0.583	0.578
Extrinsic reading motivation	Reading comprehension	−0.231	0.204	0.018	−0.58	0.066

## References

[B1-behavsci-15-00503] Abolmaali K., Mahmudi R. (2013). The prediction of academic achievement based on resilience and perception of the classroom environment. Open Science Journal of Education.

[B2-behavsci-15-00503] Aelterman N., Vansteenkiste M., Haerens L., Soenens B., Fontaine J. R. J., Reeve J. (2019). Toward an integrative and fine-grained insight in motivating and demotivating teaching styles: The merits of a circumplex approach. Journal of Educational Psychology.

[B3-behavsci-15-00503] Ainley M., Corrigan M., Richardson N. (2005). Students, tasks and emotions: Identifying the contribution of emotions to students’ reading of popular culture and popular science texts. Learning and Instruction.

[B4-behavsci-15-00503] Alexander P. A., Dai D. Y., Sternberg R. J. (2004). A model of domain learning: Reinterpreting expertise as a multidimensional, multistage process. Motivation, emotion, and cognition: Integrative perspectives on intellectual functioning and development.

[B5-behavsci-15-00503] Anttila H., Pyhalto K., Pietarinen J., Soini T. (2018). Socially embedded academic emotions in school. Journal of Education and Learning.

[B6-behavsci-15-00503] Arbuckle J. L. (2013). IBM SPSS Amos [computer program] *(Version 22.0) [Computer software]*.

[B7-behavsci-15-00503] Arhar J. M., Kromrey J. D. (1993). Interdisciplinary teaming in the middle level school: Creating a sense of belonging for at-risk middle level students. Annual Meeting of the American Educational Research Association.

[B8-behavsci-15-00503] Babad E. Y. (2009). The social psychology of the classroom.

[B9-behavsci-15-00503] Battistich V., Schaps E., Watson M., Solomon D., Lewis C. (2000). Effects of the child development project on students’ drug use and other problem behaviors. The Journal of Primary Prevention.

[B10-behavsci-15-00503] Battistich V., Solomon D., Watson M., Schaps E. (1997). Caring school communities. Educational Psychologist.

[B11-behavsci-15-00503] Becker M., McElvany N., Kortenbruck M. (2010). Intrinsic and extrinsic reading motivation as predictors of reading literacy: A longitudinal study. Journal of Educational Psychology.

[B12-behavsci-15-00503] Bryk A. S., Driscoll M. E. (1988). The school as community: Theoretical foundations, contextual influences, and consequences for students and teachers.

[B13-behavsci-15-00503] Bureau J. S., Howard J. L., Chong J. X. Y., Guay F. (2022). Pathways to student motivation: A meta-analysis of antecedents of autonomous and controlled motivations. Review of Educational Research.

[B14-behavsci-15-00503] Cain K., Oakhill J. V. (1999). Inference making ability and its relation to comprehension failure in young children. Reading and Writing: An Interdisciplinary Journal.

[B15-behavsci-15-00503] Chapman J. W., Tunmer W. E., Prochnow J. E. (2000). Early reading-related skills and performance, reading self-concept, and the development of academic self-concept: A longitudinal study. Journal of Educational Psychology.

[B16-behavsci-15-00503] Chen W., Huang Z., Peng B., Hu H. (2025). Unpacking the relationship between adolescents’ perceived school climate and negative emotions: The chain mediating roles of school belonging and social avoidance and distress. BMC Psychology.

[B17-behavsci-15-00503] Chrysochoou E. (2006). H συμβολή της εργαζόμενης μνήμης στην ακουστική κατανόηση παιδιών προσχολικής και σχολικής ηλικίας [Working memory contributions to young children’s listening comprehension]. Doctoral Dissertation.

[B18-behavsci-15-00503] Chrysochoou E., Bablekou Z. (2011). Phonological loop and central executive contributions to oral comprehension skills of 5.5 to 9.5 years old children. Applied Cognitive Psychology.

[B19-behavsci-15-00503] Chrysochoou E., Bablekou Z., Tsigilis N. (2011). Working memory contributions to reading comprehension components in middle childhood children. The American Journal of Psychology.

[B20-behavsci-15-00503] Connell J. P., Cicchetti D., Beeghly M. (1990). Context, self, and action: A motivational analysis of self-system processes across the life span. The self in transition: Infancy to childhood.

[B21-behavsci-15-00503] Connor M. C., Day S. L., Phillips B., Sparapani N., Ingebrand S. W., McLean L., Barrus A., Kaschak M. P. (2016). Reciprocal effects of self-regulation, semantic knowledge, and reading comprehension in early elementary school. Child Development.

[B22-behavsci-15-00503] Cromley J. G., Azevedo R. (2007). Testing and refining the direct and inferential mediation model of reading comprehension. Journal of Educational Psychology.

[B23-behavsci-15-00503] Daniels L. M., Stupnisky R. H., Pekrun R., Haynes T. L., Perry R. P., Newall N. E. (2009). A longitudinal analysis of achievement goals: From affective antecedents to emotional effects and achievement outcomes. Journal of Educational Psychology.

[B25-behavsci-15-00503] Deci E. L., Ryan R. M. (1985). The general causality orientations scale: Self-determination in personality. Journal of Research in Personality.

[B26-behavsci-15-00503] Deci E. L., Ryan R. M. (2000). The ‘What’ and ‘Why’ of goal pursuits: Human needs and the self-determination of behavior. Psychological Inquiry.

[B24-behavsci-15-00503] De Naeghel J., Van Keer H., Vansteenkiste M., Rosseel Y. (2012). The relation between elementary students’ recreational and academic reading motivation, reading frequency, engagement, and comprehension: A self-determination theory perspective. Journal of Educational Psychology.

[B27-behavsci-15-00503] Dermitzaki I. (2025). Fostering elementary school students’ self-regulation skills in reading comprehension: Effects on text comprehension, strategy use, and self-efficacy. Behavioral Sciences.

[B28-behavsci-15-00503] Dermitzaki I., Papakosma N. (2020). Strategies for reading comprehension in elementary school students: Their use and relations with students’ motivation and emotions. Scientific Annals-School of Psychology AUTh.

[B29-behavsci-15-00503] Dettmers S., Trautwein U., Lüdtke O., Goetz T., Frenzel A. C., Pekrun R. (2011). Students’ emotions during homework in mathematics: Testing a theoretical model of antecedents and achievement outcomes. Contemporary Educational Psychology.

[B30-behavsci-15-00503] Dewulf L., Van Braak J., Van Houtte M. (2022). Examining reading comprehension in disadvantaged segregated classes. The role of class composition, teacher trust, and teaching learning strategies. Research Papers in Education.

[B31-behavsci-15-00503] Di Leo I., Muis K. R., Singh C. A., Psaradellis C. (2019). Curiosity… confusion? frustration! The role and sequencing of emotions during mathematics problem solving. Contemporary Educational Psychology.

[B33-behavsci-15-00503] Dimitropoulou P., Filippatou D., Diakogiorgis K., Ralli A., Roussos P., Chrysochou E. (2016). The contribution of psycho-emotional factors to reading comprehension and written language production at school age. 5th Panhellenic Conference on Developmental Psychology “Putting Together the Puzzle of Human Development: Bridges with Society and Education”.

[B32-behavsci-15-00503] Dimitropoulou P., Lampropoulou A., Lykitsakou K., Chatzichristou C., Stalikas A., Triliva S., Roussi P. (2012). Ερωτηματολόγιο ’Το Σχολείο ως Κοινότητα που Νοιάζεται και Φροντίζει [Questionnaire ‘School as a caring community profile-II (SCCP-II)’ (Greek adaptation)]. Τα Ψυχομετρικά Εργαλεία στην Ελλάδα [The psychometric tools in Greece].

[B34-behavsci-15-00503] Dinsmore D. L. (2017). Toward a dynamic, multidimensional research framework for strategic processing. Educational Psychology Review.

[B35-behavsci-15-00503] Droop M., Van Elsäcker W., Voeten M. J. M., Verhoeven L. (2016). Long-term effects of strategic reading instruction in the intermediate elementary grades. Journal of Research on Educational Effectiveness.

[B36-behavsci-15-00503] Durkin D. (1993). Teaching them to read.

[B37-behavsci-15-00503] Efklides A. (2011). Interactions of metacognition with motivation and affect in self-regulated learning. The MASRL model. Educational Psychologist.

[B38-behavsci-15-00503] Efklides A., Schwartz B. L. (2024). Revisiting the metacognitive and affective model of self-regulated learning (MASRL): Origins, development, and future directions. Educational Psychology Review.

[B39-behavsci-15-00503] Elbro C., Buch-Iversen I. (2013). Activation of background knowledge for inference making: Effects on reading comprehension. Scientific Studies of Reading.

[B40-behavsci-15-00503] Ellis H. C., Ottaway S. A., Varner L. J., Becker A. S., Moore B. A. (1997). Emotion, motivation, and text comprehension: The detection of contradictions in passages. Journal of Experimental Psychology: General.

[B41-behavsci-15-00503] Ellis H. C., Varner L. J., Becker A. S., Ottaway S. A. (1995). Emotion and prior knowledge in memory and judged comprehension of ambiguous stories. Cognition & Emotion.

[B42-behavsci-15-00503] Flavell J. H. (1979). Metacognition and cognitive monitoring: A new area of cognitive–developmental inquiry. American Psychologist.

[B43-behavsci-15-00503] Flavell J. H., Weinert F., Kluwe R. (1987). Speculations about the nature and development of metacognition. Metacognition, motivation and understanding.

[B44-behavsci-15-00503] Fong Lam U., Chen W.-W., Zhang J., Liang T. (2015). It feels good to learn where I belong: School belonging, academic emotions, and academic achievement in adolescents. School Psychology International.

[B45-behavsci-15-00503] Fredrickson B. L. (2001). The role of positive emotions in positive psychology: The broaden-and-build theory of positive emotions. American Psychologist.

[B46-behavsci-15-00503] Fredrickson B. L. (2004). The broaden–and–build theory of positive emotions. Philosophical Transactions of the Royal Society of London. Series B: Biological Sciences.

[B47-behavsci-15-00503] Froiland J. M., Oros E. (2014). Intrinsic motivation, perceived competence and classroom engagement as longitudinal predictors of adolescent reading achievement. Educational Psychology.

[B48-behavsci-15-00503] Goddard R. D., Sweetland S. R., Hoy W. K. (2000). Academic emphasis of urban elementary schools and student achievement in reading and mathematics: A multilevel analysis. Educational Administration Quarterly.

[B49-behavsci-15-00503] Goetz T., Frenzel A. C., Stockinger K., Lipnevich A. A., Stempfer L., Pekrun R., Tierney R., Rizvi F., Ercikan K. (2023). Emotions in education. International encyclopedia of education.

[B50-behavsci-15-00503] Gonida E. N., Leontari A., Stalikas A., Triliva S., Roussi P. (2012). Χρήση Στρατηγικών [Learning strategies questionnaire (Greek)]. Τα Ψυχομετρικά Εργαλεία στην Ελλάδα [The psychometric tools in Greece].

[B51-behavsci-15-00503] González A., Paoloni P. V. (2014). Self-determination, behavioral engagement, disaffection, and academic performance: A mediational analysis. The Spanish Journal of Psychology.

[B52-behavsci-15-00503] Goodenow C. (1993). Classroom belonging among early adolescent students: Relationships to motivation and achievement. The Journal of Early Adolescence.

[B53-behavsci-15-00503] Graham S., Harris K. R. (2018). An examination of the design principles underlying a self-regulated strategy development study. Journal of Writing Research.

[B54-behavsci-15-00503] Graziano P. A., Reavis R. D., Keane S. P., Calkins S. D. (2007). The role of emotion regulation in children’s early academic success. Journal of School Psychology.

[B55-behavsci-15-00503] Greensfeld H., Nevo E. (2017). To use or not to use, that is the question: On students’ encounters with a library of examples. Cogent Social Sciences.

[B56-behavsci-15-00503] Guthrie J. T., Van Meter P., McCann A. D., Wigfield A., Bennett L., Poundstone C. C., Rice M. E., Faibisch F. M., Hunt B., Mitchell A. M. (1996). Growth of literacy engagement: Changes in motivations and strategies during concept-oriented reading instruction. Reading Research Quarterly.

[B57-behavsci-15-00503] Guthrie J. T., Wigfield A., Kamil M. L., Mosenthal P. B., Pearson P. D., Barr R. (2000). Engagement and motivation in reading. Handbook of reading research.

[B58-behavsci-15-00503] Guthrie J. T., Wigfield A., Lapp D., Fisher D. (2018). Literacy engagement and motivation: Rationale, research, teaching and assessment. Handbook of research on teaching the English language arts.

[B59-behavsci-15-00503] Guthrie J. T., Wigfield A., Metsala J. L., Cox K. E. (1999). Motivational and cognitive predictors of text comprehension and reading amount. Scientific Studies of Reading.

[B60-behavsci-15-00503] Guthrie J. T., Wigfield A., You W., Christenson S. L., Reschly A. L., Wylie C. (2012). Instructional contexts for engagement and achievement in reading. Handbook of research on student engagement.

[B61-behavsci-15-00503] Hagenauer G., Hascher T., Volet S. E. (2015). Teacher emotions in the classroom: Associations with students’ engagement, classroom discipline and the interpersonal teacher-student relationship. European Journal of Psychology of Education.

[B62-behavsci-15-00503] Hatzichristou C., Dimitropoulou P., Lykitsakou K., Lampropoulou A. (2020). Promoting school community well-being: Implementation of a system-level intervention program. Psychology: The Journal of the Hellenic Psychological Society.

[B63-behavsci-15-00503] Hayenga A. O., Corpus J. H. (2010). Profiles of intrinsic and extrinsic motivations: A person-centered approach to motivation and achievement in middle school. Motivation and Emotion.

[B64-behavsci-15-00503] IBM Corp (2015). IBM SPSS statistics for Windows *(Version 23.0) [Computer software]*.

[B65-behavsci-15-00503] Jang H., Kim E. J., Reeve J. (2016). Why students become more engaged or more disengaged during the semester: A self-determination theory dual-process model. Learning and Instruction.

[B66-behavsci-15-00503] Jang L. Y., Liu W. C. (2012). 2 × 2 Achievement goals and achievement emotions: A cluster analysis of students’ motivation. European Journal of Psychology of Education.

[B67-behavsci-15-00503] Kendeou P., McMaster K. L., Christ T. J. (2016). Reading comprehension: Core components and processes. Policy Insights from the Behavioral and Brain Sciences.

[B68-behavsci-15-00503] Kim C., Pekrun R., Spector J. M., Merrill M. D., Elen J., Bishop M. J. (2014). Emotions and motivation in learning and performance. Handbook of research on educational communications and technology.

[B69-behavsci-15-00503] King R. B., Gaerlan M. J. M. (2014). High self-control predicts more positive emotions, better engagement, and higher achievement in school. European Journal of Psychology of Education.

[B70-behavsci-15-00503] Kintsch W., Tobias S., Duffy T. M. (2009). Learning and constructivism. Constructivist instruction: Success or failure?.

[B71-behavsci-15-00503] Lau K. (2009). Reading motivation, perceptions of reading instruction and reading amount: A comparison of junior and senior secondary students in Hong Kong. Journal of Research in Reading.

[B72-behavsci-15-00503] Liao X., Zhu X., Zhao P. (2022). The mediating effects of reading amount and strategy use in the relationship between intrinsic reading motivation and comprehension: Differences between Grade 4 and Grade 6 students. Reading and Writing.

[B73-behavsci-15-00503] Lickona T., Davidson M., Stalikas A., Triliva S., Roussi P. (2012). School as a caring community profile-II (SCCP-II): A survey of students, staff, and parents scale descriptions. Psychometric tools in Greece.

[B74-behavsci-15-00503] Linnenbrink E. A. (2007). The role of affect in student learning. Emotion in education.

[B75-behavsci-15-00503] Logan J. A. R., Piasta S. B., Justice L. M., Schatschneider C., Petrill S. (2011). Children’s attendance rates and quality of teacher-child interactions in at-risk preschool classrooms: Contribution to children’s expressive language growth. Child & Youth Care Forum.

[B76-behavsci-15-00503] Lohbeck A., Moschner B. (2022). Motivational regulation strategies, academic self-concept, and cognitive learning strategies of university students: Does academic self-concept play an interactive role?. European Journal of Psychology of Education.

[B77-behavsci-15-00503] Magi K., Mannamaa M., Kikas E. (2016). Profiles of self-regulation in elementary grades: Relations to math and reading skills. Learning & Individual Differences.

[B78-behavsci-15-00503] Magnusson C. G., Roe A., Blikstad-Balas M. (2019). To what extent and how are reading comprehension strategies part of language arts instruction? A study of lower secondary classrooms. Reading Research Quarterly.

[B79-behavsci-15-00503] Mason L. H. (2013). Teaching students who struggle with learning to think before, while, and after reading: Effects of self-regulated strategy development instruction. Reading & Writing Quarterly.

[B80-behavsci-15-00503] Maxwell S., Reynolds K. J., Lee E., Subasic E., Bromhead D. (2017). The impact of school climate and school identification on academic achievement: Multilevel modeling with student and teacher data. Frontiers in Psychology.

[B81-behavsci-15-00503] McDaniel M. A., Einstein G. O. (2000). Strategic and automatic processes in prospective memory retrieval: A multiprocess framework. Applied Cognitive Psychology.

[B82-behavsci-15-00503] McMillan D. W., Chavis D. M. (1986). Sense of community: A definition and theory. Journal of Community Psychology.

[B83-behavsci-15-00503] Meece J. L., Blumenfeld P. C., Hoyle R. H. (1988). Students’ goal orientations and cognitive engagement in classroom activities. Journal of Educational Psychology.

[B84-behavsci-15-00503] Mega C., Ronconi L., De Beni R. (2014). What makes a good student? How emotions, self-regulated learning, and motivation contribute to academic achievement. Journal of Educational Psychology.

[B85-behavsci-15-00503] Mokhtari K., Reichard C. A. (2002). Assessing students’ metacognitive awareness of reading strategies. Journal of Educational Psychology.

[B86-behavsci-15-00503] Moore A. L. (2015). Reading comprehension: A research review of cognitive skills, strategies, and interventions.

[B87-behavsci-15-00503] Morgan P. L., Fuchs D. (2007). Is there a bidirectional relationship between children’s reading skills and reading motivation?. Exceptional Children.

[B88-behavsci-15-00503] Mouratidis A., Michou A. (2011). Self-determined motivation and social achievement goals in children’s emotions. Educational Psychology.

[B89-behavsci-15-00503] Mucherah W., Yoder A. (2008). Motivation for reading and middle school students’ performance on standardized testing in reading. Reading Psychology.

[B90-behavsci-15-00503] Muijselaar M. L., Swart N. M., Steenbeek-Planting E. G., Droop M., Verhoeven L., de Jong P. F. (2017). Developmental relations between reading comprehension and reading strategies. Scientific Studies of Reading.

[B91-behavsci-15-00503] Multon K. D., Brown S. D., Lent R. W. (1991). Relation of self-efficacy beliefs to academic outcomes: A meta-analytic investigation. Journal of Counseling Psychology.

[B92-behavsci-15-00503] Naceur A., Schiefele U. (2005). Motivation and learning—The role of interest in construction of representation of text and long-term retention: Inter- and intraindividual analyses. European Journal of Psychology of Education.

[B93-behavsci-15-00503] Nejadihassan S., Arabmofrad A. (2016). A review of relationship between self-regulation and reading comprehension. Theory and Practice in Language Studies.

[B94-behavsci-15-00503] Nolen S. B., Haladyna T. M. (1990). Personal and environmental influences on students’ beliefs about effective study strategies. Contemporary Educational Psychology.

[B95-behavsci-15-00503] Oakhill J. (1984). Inferential and memory skills in children’s comprehension of stories. British Journal of Educational Psychology.

[B96-behavsci-15-00503] Obergriesser S., Stoeger H. (2020). Students’ emotions of enjoyment and boredom and their use of cognitive learning strategies—How do they affect one another?. Learning and Instruction.

[B97-behavsci-15-00503] Paoloni P. V. R. (2014). Emotions in academic contexts: Theoretical perspectives and implications for educational practice in college. Electronic Journal of Research in Educational Psychology.

[B98-behavsci-15-00503] Pardo L. S. (2004). What every teacher needs to know about comprehension. The Reading Teacher.

[B99-behavsci-15-00503] Peixoto F., Mata L., Monteiro V., Sanches C., Pekrun R. (2015). The achievement emotions questionnaire: Validation for pre-adolescent students. European Journal of Developmental Psychology.

[B100-behavsci-15-00503] Pekrun R. (1992). The impact of emotions on learning and achievement: Towards a theory of cognitive/motivational mediators. Applied Psychology.

[B101-behavsci-15-00503] Pekrun R. (2000). A social-cognitive, control-value theory of achievement emotions. Advances in psychology.

[B102-behavsci-15-00503] Pekrun R. (2006). The control-value theory of achievement emotions: Assumptions, corollaries, and implications for educational research and practice. Educational Psychology Review.

[B103-behavsci-15-00503] Pekrun R., Patulny R., Bellocchi A., Olson R. E., Khorana S., McKenzie J., Peterie M. (2019). Achievement emotions: A control-value theory perspective. Emotions in late modernity.

[B104-behavsci-15-00503] Pekrun R. (2022). Emotions in reading and learning from texts: Progress and open problems. Discourse Processes.

[B105-behavsci-15-00503] Pekrun R. (2024). Control-value theory: From achievement emotion to a general theory of human emotions. Educational Psychology Review.

[B106-behavsci-15-00503] Pekrun R., Elliot A. J., Maier M. A. (2006). Achievement goals and discrete achievement emotions: A theoretical model and prospective test. Journal of Educational Psychology.

[B107-behavsci-15-00503] Pekrun R., Frenzel A. C., Goetz T., Perry R. P. (2007). The control-value theory of achievement emotions. Emotion in education.

[B108-behavsci-15-00503] Pekrun R., Goetz T., Frenzel A. C., Barchfeld P., Perry R. P. (2011). Measuring emotions in students’ learning and performance: The Achievement Emotions Questionnaire (AEQ). Contemporary Educational Psychology.

[B109-behavsci-15-00503] Pekrun R., Goetz T., Perry R. P., Kramer K., Hochstadt M., Molfenter S. (2004). Beyond test anxiety: Development and validation of the test emotions questionnaire (TEQ). Anxiety, Stress & Coping.

[B110-behavsci-15-00503] Pekrun R., Goetz T., Titz W., Perry R. P. (2002). Academic emotions in students’ self-regulated learning and achievement: A program of qualitative and quantitative research. Educational Psychologist.

[B111-behavsci-15-00503] Pekrun R., Hall N. C., Goetz T., Perry R. P. (2014). Boredom and academic achievement: Testing a model of reciprocal causation. Journal of Educational Psychology.

[B112-behavsci-15-00503] Pekrun R., Marsh H. W., Elliot A. J., Stockinger K., Perry R. P., Vogl E., Goetz T., van Tilburg W. A. P., Lüdtke O., Vispoel W. P. (2023). A three-dimensional taxonomy of achievement emotions. Journal of Personality and Social Psychology.

[B113-behavsci-15-00503] Pekrun R., Perry R. P., Pekrun R., Linnenbrink-Garcia L. (2014). Control-value theory of achievement emotions. International handbook of emotions in education.

[B114-behavsci-15-00503] Pinheiro A. P., Del Re E., Nestor P. G., McCarley R. W., Gonçalves Ó. F., Niznikiewicz M. (2013). Interactions between mood and the structure of semantic memory: Event-related potentials evidence. Social Cognitive and Affective Neuroscience.

[B115-behavsci-15-00503] Pintrich P. R. (2002). The role of metacognitive knowledge in learning, teaching, and assessing. Theory Into Practice.

[B116-behavsci-15-00503] Pintrich P. R. (2004). A conceptual framework for assessing motivation and self-regulated learning in college students. Educational Psychology Review.

[B117-behavsci-15-00503] Pressley M., Afflerbach P. (1995). Verbal protocols of reading: The nature of constructively responsive reading.

[B118-behavsci-15-00503] Pressley M., Gaskins I. W. (2006). Metacognitively Competent Reading Comprehension Is Constructively Responsive Reading: How Can Such Reading Be Developed in Students?. Metacognition and Learning.

[B119-behavsci-15-00503] Putwain D. W., Sander P., Larkin D. (2013). Academic self-efficacy in study-related skills and behaviours: Relations with learning-related emotions and academic success. British Journal of Educational Psychology.

[B120-behavsci-15-00503] Putwain D. W., Schmitz E. A., Wood P., Pekrun R. (2021). The role of achievement emotions in primary school mathematics: Control–value antecedents and achievement outcomes. British Journal of Educational Psychology.

[B121-behavsci-15-00503] Ranellucci J., Hall N. C., Goetz T. (2015). Achievement goals, emotions, learning, and performance: A process model. Motivation Science.

[B122-behavsci-15-00503] Reeve J., Cheon S. H. (2021). Autonomy-supportive teaching: Its malleability, benefits, and potential to improve educational practice. Educational Psychologist.

[B123-behavsci-15-00503] Retelsdorf J., Köller O., Möller J. (2011). On the effects of motivation on reading performance growth in secondary school. Learning and Instruction.

[B124-behavsci-15-00503] Roberts W., Horn A., Battistich V. (1995). Assessing students’ and teachers’ sense of the school as a caring community. Annual Meeting of the American Educational Research Association.

[B125-behavsci-15-00503] Rubie-Davies C., Fives H., Gill M. G. (2014). Teachers’ instructional beliefs and the classroom climate. International handbook of research on teachers’ beliefs.

[B126-behavsci-15-00503] Rubie-Davies C., Asil M., Teo T. (2016). Assessing measurement invariance of the student personal perception of classroom climate across different ethnic groups. Journal of Psychoeducational Assessment.

[B127-behavsci-15-00503] Ryan R. M., Deci E. L. (2000). Self-determination theory and the facilitation of intrinsic motivation, social development, and well-being. American Psychologist.

[B128-behavsci-15-00503] Ryan R. M., Deci E. L. (2020). Intrinsic and extrinsic motivation from a self-determination theory perspective: Definitions, theory, practices, and future directions. Contemporary Educational Psychology.

[B129-behavsci-15-00503] Samuelstuen M. S., Bråten I. (2005). Decoding, knowledge, and strategies in comprehension of expository text. Scandinavian Journal of Psychology.

[B130-behavsci-15-00503] Scherer K. R. (2009). The dynamic architecture of emotion: Evidence for the component process model. Cognition & Emotion.

[B131-behavsci-15-00503] Schiefele U., Pieters J. M., Breuer K., Simons P. R.-J. (1990). The influence of topic interest, prior knowledge, and cognitive capabilities on text comprehension. Learning environments: Contributions from Dutch and German research.

[B132-behavsci-15-00503] Schiefele U., Wentzel K. R., Wigfield A. (2009). Situational and individual interest. Handbook of motivation at school.

[B133-behavsci-15-00503] Schiefele U., Schaffner E., Möller J., Wigfield A. (2012). Dimensions of reading motivation and their relation to reading behavior and competence. Reading Research Quarterly.

[B134-behavsci-15-00503] Schiefele U., Stutz F., Schaffner E. (2016). Longitudinal relations between reading motivation and reading comprehension in the early elementary grades. Learning and Individual Differences.

[B136-behavsci-15-00503] Schunk D. H. (1991). Self-efficacy and academic motivation. Educational Psychologist.

[B137-behavsci-15-00503] Schunk D. H., Pintrich P. R., Meece J. L., Makris N., Pneumatikos D., Koulentianou M. (2010). Τα κίνητρα στην εκπαίδευση [Greek].

[B138-behavsci-15-00503] Schunk D. H., Zimmerman B. J., Weiner I. (2012). Self-regulation and learning. Handbook of psychology, educational psychology.

[B139-behavsci-15-00503] Schutz P. A., Pekrun R. (2007). Emotion in education.

[B135-behavsci-15-00503] Schünemann N., Spörer N., Brunstein J. C. (2013). Integrating self-regulation in whole-class reciprocal teaching: A moderator–mediator analysis of incremental effects on fifth graders’ reading comprehension. Contemporary Educational Psychology.

[B140-behavsci-15-00503] Scrimin S., Mason L. (2015). Does mood influence text processing and comprehension? Evidence from an eye-movement study. British Journal of Educational Psychology.

[B141-behavsci-15-00503] Sedek G., Von Hecker U. (2004). Effects of subclinical depression and aging on generative reasoning about linear orders: Same or different processing limitations?. Journal of Experimental Psychology: General.

[B142-behavsci-15-00503] Shochet I. M., Dadds M. R., Ham D., Montague R. (2006). School connectedness is an underemphasized parameter in adolescent mental health: Results of a community prediction study. Journal of Clinical Child & Adolescent Psychology.

[B143-behavsci-15-00503] Solomon D., Watson M. S., Delucchi K. L., Schaps E., Battistich V. (1988). Enhancing children’s prosocial behavior in the classroom. American Educational Research Journal.

[B144-behavsci-15-00503] Spörer N., Schünemann N. (2014). Improvements of self-regulation procedures for fifth graders’ reading competence: Analyzing effects on reading comprehension, reading strategy performance, and motivation for reading. Learning and Instruction.

[B145-behavsci-15-00503] Stanovich K. E. (1986). Matthew Effects in Reading: Some Consequences of Individual Differences in the Acquisition of Literacy. Reading Research Quarterly.

[B146-behavsci-15-00503] Stutz F., Schaffner E., Schiefele U. (2016). Relations among reading motivation, reading amount, and reading comprehension in the early elementary grades. Learning and Individual Differences.

[B147-behavsci-15-00503] Sun Y., Wang J., Dong Y., Zheng H., Yang J., Zhao Y., Dong W. (2021). The relationship between reading strategy and reading comprehension: A meta-analysis. Frontiers in Psychology.

[B148-behavsci-15-00503] Swan M. (2008). Talking sense about learning strategies. RELC Journal.

[B149-behavsci-15-00503] Sweeny K., Vohs K. D. (2012). On near misses and completed tasks: The nature of relief. Psychological Science.

[B150-behavsci-15-00503] Šabec T. (2022). Reading motivation and reading strategies in the primary school classroom. European Journal of Literature, Language and Linguistics Studies.

[B151-behavsci-15-00503] Taboada A., Tonks S. M., Wigfield A., Guthrie J. T. (2009). Effects of motivational and cognitive variables on reading comprehension. Reading and Writing.

[B152-behavsci-15-00503] Tan J., Mao J., Jiang Y., Gao M. (2021). The influence of academic emotions on learning effects: A systematic review. International Journal of Environmental Research and Public Health.

[B153-behavsci-15-00503] Taylor G., Jungert T., Mageau G. A., Schattke K., Dedic H., Rosenfield S., Koestner R. (2014). A self-determination theory approach to predicting school achievement over time: The unique role of intrinsic motivation. Contemporary Educational Psychology.

[B154-behavsci-15-00503] Thapa A., Cohen J., Guffey S., Higgins-D’Alessandro A. (2013). A review of school climate research. Review of Educational Research.

[B155-behavsci-15-00503] Toste J. R., Didion L., Peng P., Filderman M. J., McClelland A. M. (2020). A meta-analytic review of the relations between motivation and reading achievement for K–12 students. Review of Educational Research.

[B156-behavsci-15-00503] Trevors G. J., Muis K. R., Pekrun R., Sinatra G. M., Muijselaar M. M. L. (2017). Exploring the relations between epistemic beliefs, emotions, and learning from texts. Contemporary Educational Psychology.

[B157-behavsci-15-00503] Trevors G. J., Muis K. R., Pekrun R., Sinatra G. M., Winne P. H. (2016). Identity and epistemic emotions during knowledge revision: A potential account for the backfire effect. Discourse Processes.

[B158-behavsci-15-00503] Turner J. E., Schallert D. L. (2001). Expectancy–value relationships of shame reactions and shame resiliency. Journal of Educational Psychology.

[B159-behavsci-15-00503] Tyng C. M., Amin H. U., Saad M. N. M., Malik A. S. (2017). The influences of emotion on learning and memory. Frontiers in Psychology.

[B160-behavsci-15-00503] Tze V. M. C., Klassen R. M., Daniels L. M. (2014). Patterns of boredom and its relationship with perceived autonomy support and engagement. Contemporary Educational Psychology.

[B161-behavsci-15-00503] Urdan T., Schoenfelder E. (2006). Classroom effects on student motivation: Goal structures, social relationships, and competence beliefs. Journal of School Psychology.

[B162-behavsci-15-00503] Urke H. B., Kristensen S. M., Bøe T., Gaspar De Matos M., Wiium N., Årdal E., Larsen T. (2023). Perceptions of a caring school climate and mental well-being: A one-way street? Results from a random intercept cross-lagged panel model. Applied Developmental Science.

[B163-behavsci-15-00503] Van Den Berghe L., Soenens B., Vansteenkiste M., Aelterman N., Cardon G., Tallir I. B., Haerens L. (2013). Observed need-supportive and need-thwarting teaching behavior in physical education: Do teachers’ motivational orientations matter?. Psychology of Sport and Exercise.

[B164-behavsci-15-00503] Van Den Broek P., Espin C. A. (2012). Connecting cognitive theory and assessment: Measuring individual differences in reading comprehension. School Psychology Review.

[B165-behavsci-15-00503] Vansteenkiste M., Lens W., Deci E. L. (2006). Intrinsic versus extrinsic goal contents in Self-Determination Theory: Another look at the quality of academic motivation. Educational Psychologist.

[B166-behavsci-15-00503] Villavicencio F. T., Bernardo A. B. I. (2013). Positive academic emotions moderate the relationship between self-regulation and academic achievement. British Journal of Educational Psychology.

[B167-behavsci-15-00503] Von Hecker U., Meiser T. (2005). Defocused attention in depressed mood: Evidence from source monitoring. Emotion.

[B168-behavsci-15-00503] Wang J. H., Guthrie J. T. (2004). Modeling the effects of intrinsic motivation, extrinsic motivation, amount of reading, and past reading achievement on text comprehension between U.S. and Chinese students. Reading Research Quarterly.

[B169-behavsci-15-00503] Wang M.-T., Degol J. L. (2016). School Climate: A review of the construct, measurement, and impact on student outcomes. Educational Psychology Review.

[B170-behavsci-15-00503] Wang M.-T., Holcombe R. (2010). Adolescents’ perceptions of school environment, engagement, and academic achievement in middle school. American Educational Research Journal.

[B171-behavsci-15-00503] Wang W., Vaillancourt T., Brittain H. L., McDougall P., Krygsman A., Smith D., Cunningham C. E., Haltigan J. D., Hymel S. (2014). School climate, peer victimization, and academic achievement: Results from a multi-informant study. School Psychology Quarterly.

[B172-behavsci-15-00503] Wei L. S., Elias H. (2011). Relationship between students’ perceptions of classroom enviroment and their motivation in learning english language. International Journal of Humanities and Social Science.

[B173-behavsci-15-00503] Weiner B. (1985). An attributional theory of achievement motivation and emotion. Psychological Review.

[B174-behavsci-15-00503] Weinstein C. E., Acee T. W., Jung J. (2011). Self-regulation and learning strategies. New Directions for Teaching and Learning.

[B175-behavsci-15-00503] Weinstein C. E., Mayer R. E., Wittrock M. (1986). The teaching of learning strategies. Handbook of research on teaching.

[B176-behavsci-15-00503] Wigfield A., Guthrie J. T. (1997). Relations of children’s motivation for reading to the amount and breadth or their reading. Journal of Educational Psychology.

[B177-behavsci-15-00503] Wigfield A., Guthrie J. T., Meece J. L., Eccles J. S. (2010). The impact of concept-oriented reading instruction on students’ reading motivation, reading engagement, and reading comprehension. Handbook on schools, schooling, and human development.

[B178-behavsci-15-00503] Williams G. C., Deci E., Stalikas A., Triliva S., Roussi P. (2012). The learning climate questionnaire (LCQ). (Adaptation: Dimitropoulou, P., Lykitsakou, K., & Chatzichristou, C.). Psychometric tools in Greece.

[B179-behavsci-15-00503] Williams G. C., Deci E. L. (1996). Internalization of biopsychosocial values by medical students: A test of self-determination theory. Journal of Personality and Social Psychology.

[B180-behavsci-15-00503] Zaccoletti S., Altoè G., Mason L. (2020). The interplay of reading-related emotions and updating in reading comprehension performance. British Journal of Educational Psychology.

[B181-behavsci-15-00503] Zahid G. (2014). Direct and indirect impact of perceived school climate upon student outcomes. Asian Social Science.

[B182-behavsci-15-00503] Zeidner M. (2007). Test anxiety in educational contexts. Emotion in education.

[B183-behavsci-15-00503] Zimmerman B. J., Boekaerts M., Pintrich P. R., Zeidner M. (2000). Attaining self-regulation: A social-cognitive perspective. Handbook of self-regulation.

